# Dataset on humanic clues and customer loyalty in selected hospitals in Lagos State, Nigeria

**DOI:** 10.1016/j.dib.2018.06.079

**Published:** 2018-07-04

**Authors:** Taiye Borishade, Rowland E. Worlu, Oladele Kehinde, Olaleke Ogunnaike, Oluwole Iyiola, Joy Dirisu, Maxwell Olokundun, Ayodotun Ibidunni

**Affiliations:** Covenant University, Nigeria

**Keywords:** Humanic clues, Customer, Loyalty, Healthcare, Nigeria

## Abstract

This article describes survey result on humanic clues and customer loyalty in selected hospitals in Lagos State, Nigeria. This paper obtains information from the customer experience management strategy in considering the techniques in which customer loyalty can be built. 365 copies of questionnaires were retrieved from the customers of the selected four private hospitals in Lagos State. The data gathered from the survey customers were subjected to inferential and descriptive statistics in order to ascertain the sum, mean, standard deviation and the relative importance index (RII). The retrieved copies of questionnaires were analysed utilising SPSS (22). Using the Categorical Regression CATREG analysis, the data article establish that humanic clues have positive influence on customer loyalty. The data collected is openly presented to enhance further analysis.

**Specification Table**

**Table:** 

**Subject area**	Marketing, Management
**More Specific Subject Area**	Healthcare Marketing
**Type of Data**	Table
**How Data was Acquired**	Researcher-made survey analysis
**Data format**	Inferential, Descriptive and statistical data
**Experimental Factors**	The respondents for this study are the healthcare customers of four private hospitals adjudged to be the best in Nigeria.
**Experimental features**	The survey customers responded to the questionnaire on the influence of humanic clues and loyalty.
**Data source location**	Lagos State, Nigeria
**Data Accessibility**	Data are enclosed within this study.

**Value of the data**

• These article made available data on the influence of humanic clues on customer loyalty among healthcare customers of Nigeria. However, to enhance the generalization of result, other scholars may choose to replicate this study in a different context [5].
• Future studies can focus on the other variables of the customer experience management strategies [6].
• Looking at scarce data on how customer loyalty can be built. Further studies can focus on the functional and mechanic clues alongside the humanic clues [9].

## 1 Data

The data encompassed the fresh statistical data on the influence of humanic clues on customer loyalty in the healthcare segment of Nigeria. The data show result of the descriptive statistics of the influence of humanic clues on customer׳s loyalty based on the views of the healthcare customers. Using the relative importance index or RII formula. The Relative Importance Index (RII) for each of the variables used to investigate the influence of humanic clues on customer loyalty was computed.

$$RII=\left(\sum_{i=1}^{5}W_{l}\times f_{xi}\right)\;N(A)$$

where,

w=The weight age of the respondents
A=1, 2, 3, 4, 5
f_xi_=The frequency of every respondent
N=Total number of respondents [3], [8]

According to several scholars, the standard deviations and mean are not dependable measurements for weighing overall ranking of the elements [1]. The Relative Importance Index (RII) provides a descriptive interpretation of the most important element [2]. Therefore, in this study, the RII was considered appropriate in examining which components of the humanic clues are ranked highest in building customer loyalty of healthcare services among the customers of the four private hospitals investigated (Table 1).

**Table 1:** The influence of humanic clues on customer loyalty.

**Components of Humanic Clues**	**N**	**Sum**	**Relative importance index**	**Rank**
The service provider׳s neatness	365	1512	0.828	1
The friendly actions of service providers	365	1509	0.826	2
The respect and courtesy from the health care service providers	365	1488	0.815	3
Caring expression of the service providers	365	1486	0.814	4
Service provider׳s understanding	365	1480	0.811	5
The mindfulness of the service providers	365	1455	0.797	6
The tone of voice	365	1449	0.794	7
The responsiveness of service providers	365	1419	0.778	8
The body language of the service providers	365	1367	0.749	9

The result in Table 2 shows the responses by the male and female customers on how humanic clues influence customer loyalty. From the result, it is evident that both the male and female customers have similar responses on the influence of humanic clues and customer loyalty. The result revealed positive responses concerning all the statements related to the influence of humanic clues and customer loyalty as all the mean scores of the variables investigated are more than the 3.01 on a 5-likert type scale.

**Table 2:** The influence of humanic clues on customer loyalty based on gender.

**Components of humanic Clues and customer loyalty**	**Mean Scores for Males**	**Mean Scores for Females**
The friendly actions of the provider of service.	4.15	4.14
The caring expression of the providers of service.	4.03	4.10
The tone of voice of the health care service provider pleases me.	3.82	4.11
The body language of the health care service provider.	3.75	3.75
The respect and courtesy from the health care service provider.	4.13	4.03
The neatness of the health care service provider fascinates me.	4.09	4.19
The responsiveness of the provider.	3.85	3.92
The mindfulness of the provider.	3.94	4.02
The understanding of the health care service provider.	4.10	4.02

### 1.1 Test of hypothesis

From the result presented in Table 3, the R Square tells the extent of the modification in the dependent variable (Customer loyalty) is explicated by the model (Humanic Clues: the respect and courtesy, tone of voice, friendly actions, body language, caring expression). Here, the R Square value of .504 is stated as a percentage; this implies that the model (humanic clues) elucidates 50.4% of the variance on customer loyalty. This is very reasonable result when compare to some outcome that are reported in the journals [4], [7], [9].

**Table 3:** Regression effects of humanic clues on customer loyalty. Source: Field Survey, 2017

	Standardized Coefficients		df	F	Sig.
	Beta	Bootstrap (1000) Estimate of Std. Error			

The friendly actions of the health care service providers.	.202	.075	3	7.358	.000
The caring expression of the health care service providers.	.172	.067	2	6.578	.002
The tone of voice of the health care service provider.	.073	.075	3	.931	.426
The body language of the health care service provider.	.215	.082	3	6.836	.000
The respect and courtesy from the health care service provider.	.285	.070	3	16.462	.000

R	.710			
R^2^	.504			
Adj. R^2^	.484			
F	25.211			
Overall Sig.	.000			

Furthermore, the result of the model in Table 3, shows that the respect and courtesy of the healthcare service provider having the highest beta value of (*β*=.285) contribute mostly to explaining the influence of humanic clues on customer loyalty of healthcare services. This is followed by the body language of the healthcare service provider (*beta*=.215), the friendly actions of the service providers (*beta*=.202) and the caring expression of the service providers scale (*beta*=.172). This means that the healthcare customers of the organisations believe that the respect and courtesy of the healthcare service provider makes the strongest unique contribution to explaining the loyalty of customers. This findings corroborate [9] findings.

## 2 Experimental design, materials and methods

### 2.1 Data characteristics

The data for this article are quantitative in nature. The quantitative data are mainly the socio-economic characteristics of customers and healthcare experts sampled, as well as their perception of the extent to which humanic clues influence customer loyalty of the customers in the four hospitals sampled.

### 2.2 Data source

The data for this objective were derived from the selected private healthcare customers of the hospitals in Lagos, Nigeria. The data from the healthcare customers on the extent to which humanic clues affect the loyalty of customers were sourced via the administration of structured questionnaire.

The copies of questionnaires were distributed to the healthcare customers of the four private hospitals during the working hours of the week days. Accidental or convenience sampling technique was adopted in dispensing the questionnaire to the healthcare customers during the waiting time of the week days. Although, four hundred (400) questionnaires comprising 100 questionnaires administered to the customers of each of the four private hospitals were distributed. However, 365 copies of questionnaires representing 91.25% of the questionnaire disseminated were salvaged.

### 2.3 Data analysis

The extent to which humanic clues affect the loyalty of customers was investigated by asking the respondents to indicate the degree of agreement with the statement relating to the objective based on a 5-point Likert scale, where 5=strongly agree, 4=agree, 3=undecided, 2=disagree and 1=strongly disagree. The data were subjected to descriptive statistics, and this was used to compute the sum, mean, standard deviation and the relative importance index of the variables used in assessing humanic clues. Tables were used to present the results, the frequencies and percentages to provide adequate understanding of the respondents’ characteristics and their view on the extent to which humanic clues affect the loyalty of customers. CATREG in SPSS was also used to investigate the influence of humanic clues on the loyalty of customers in the four private hospitals sampled.
