# Supplementary material for: Dataset on humanic clues and customer loyalty in selected hospitals in Lagos State, Nigeria
Source: Data Brief. 2018 Jul 4;19:1948–52. doi: 10.1016/j.dib.2018.06.079 (PMC6141372; doi:10.1016/j.dib.2018.06.079)
Supplement: Supplementary file 3 — Supplementary material. [file mmc3.docx]

**CHAPTER FOUR**

**RESULTS**

**4.0 Preamble**

This chapter starts with the interpretation of data based on the responses recorded in the questionnaires distributed to the respondents, followed by the description of the respondents’ demographic data using tables. The hypotheses formulated for this research informed the organisation of the findings. Each hypothesis is focused on the variables identified i.e. using (customer loyalty as the dependent variable and customer experience management as the independent variable). The result of the analysis of each of the hypothesis was interpreted; and where necessary, the findings from the demographical data were employed to buttress and compare the findings of the hypotheses stated in this thesis.

## **4.1 Presentation of Data**

The data gathered for this study were presented using tables showing relevant information on the total number of questionnaires distributed and retrieved from the respondents. Details of these can be seen in Table 4.1a.

## **4.1.1 The Response Rate of the Survey**

Data for the first phase were collected from customers of the four private hospitals in Lagos Metropolis considered to be the best private hospitals in Nigeria (Toriola, 2014). The data were collected via questionnaire in the four private hospitals. A total of four hundred questionnaires were administered to the customers of the four hospitals, that is, one hundred (100) questionnaires for each of the hospital to confirm whether customer experience management strategies can actually lead to customer loyalty. A total of 365 copies were retrieved and found to be valid and were used in the analysis. The number of questionnaires useable represents 91.25% response rate. The high response rate was due to the continuous visit and several calls made by the researcher to the respondents and probably because of the high interest the sampled respondents had in the study. This response rate is deemed reasonably high when compared to the response rate employed by previous studies. For example Nwokah and Nwokah (2013) examined the delivery of customer experience management practices in the UK and Nigeria Aviation Industry and of the 500 copies of questionnaires administered in that study 406 were retrieved, representing (81.2%).

**Table 4.1: Response rate of the questionnaires administered and retrieved from the customers**

| S/N | Hospitals | Questions Distributed | Num Retrieved and analyzed | Num. not Retrieved | Response Rate (%) |
| --- | --- | --- | --- | --- | --- |
| 1 | Lagoon Hospital (LGH) | 100 | 91 | 9 | 22.75 |
| 2 | Reddington Hospital (RDTH) | 100 | 89 | 11 | 22.25 |
| 3 | Eko Hospital (EKOH) | 100 | 92 | 8 | 23 |
| 4 | St.Nicholas Hospital (STNH) | 100 | 93 | 7 | 23.25 |
|  | Total | 400 | 365 | 35 | 91.25 |

Source: Researcher’s Field Survey, 2016

## **4.2 Data Analysis and Interpretation**

|  | LGH | RDTH | EKOH | STNH | Total |
| --- | --- | --- | --- | --- | --- |
| Number of target respondents | 100 | 100 | 100 | 100 | 400 |
| Share of actual respondents | 91 | 89 | 92 | 93 | 365 |
| Share of response rate (%) | 91 | 89 | 92 | 93 | 91.25 |
| Share of Male Respondents (in %) | 42.9 | 39.3 | 44.6 | 49.5 | 44.1 |
| Share of Female Respondents (in %) | 57.1 | 60.7 | 55.4 | 50.5 | 55.9 |
| Average age of the respondents in years (in %) | 64.8 | 38.2 | 51.1 | 38.7 | 42.2 |
| Respondents who completed a University education (in %) | 82.4 | 69.7 | 42.4 | 64.5 | 64.7 |
| Respondents who are married (in %) | 62.6 | 64.0 | 78.3 | 67.7 | 68.2 |
| Respondents by patronage experience | 56.0 | 69.7 | 69.6 | 46.2 | 60.3 |

**Table 4.2: Sample Characteristics of Healthcare Customers**

Source: Researcher’s Field Survey, 2016

KEY: LGH = Lagoon Hospital; RDTH= Reddington Hospital,

EKOH = EKO Hospital; STNH = St. Nicholas Hospital

##

## **Table 4.2.1: Demographic Characteristics of the Healthcare Customers in the four Hospitals**

| **GENDER** | **Frequency** | **Percent** |
| --- | --- | --- |
| Male | 161 | 44.1 |
| Female | 204 | 55.9 |
| Total | 365 | 100.0 |
| **AGE** |  |  |
| 18-30 | 102 | 27.9 |
| 31-40 | 176 | 48.2 |
| 41-50 | 70 | 19.2 |
| 51-ABOVE | 17 | 4.7 |
| Total | 365 | 100.0 |
| **Highest Educational Qualification** | | |
| O’ Level WAEC, GCE | 39 | 10.7 |
| B.Sc. | 236 | 64.7 |
| M.Sc/MBA | 67 | 18.4 |
| OTHERS | 23 | 6.3 |
| Total | 365 | 100.0 |
| **Marital Status** | | |
| Single | 110 | 30.1 |
| Married | 249 | 68.2 |
| Divorced/separated | 6 | 1.6 |
| Total | 365 | 100.0 |
| **Occupation** | | |
| Student | 95 | 26.0 |
| Employer | 67 | 18.4 |
| Employee | 203 | 55.6 |
| Total | 365 | 100.0 |
| **Type of Customer** | | |
| Corporate Customer | 216 | 59.2 |
| Private Individual | 149 | 40.8 |
| Total | 365 | 100.0 |
| **Hospital Patronized** | | |
| Lagoon Hospital | 91 | 24.9 |
| Reddington Hospital | 89 | 24.4 |
| EKO Hospital | 92 | 25.2 |
| St. Nicholas Hospital | 93 | 25.5 |
| Total | 365 | 100.0 |
| **Years of Patronage Experience** | | |
| 1 - 5 years | 220 | 60.3 |
| 6 – 10 years | 106 | 29.0 |
| 11 – 15 years | 22 | 6.0 |
| 16 years and above | 17 | 4.7 |
| Total | 365 | 100.0 |

Source: Researcher’s Field Survey, 2016

|  | | | | | | | |
| --- | --- | --- | --- | --- | --- | --- | --- |
| **Table 4.2.1a: Gender and Hospital Patronized** | | | | | | | |
|  | | | Hospitals Patronized | | | | Total n(%) |
|  |  |  | Lagoon Hospital | Reddington Hospital | EKO Hospital | St. Nicholas Hospital |  |
| Gender | MALE | Count | 39 | 35 | 41 | 46 | 161 |
|  |  | % within Gender | 24.2 | 21.7 | 25.5 | 28.6 | 100.0 |
|  |  | % within Which of the hospitals do you patronize? | 42.9 | 39.3 | 44.6 | 49.5 | 44.1 |
|  |  | % of Total | 10.7 | 9.6 | 11.2 | 12.6 | 44.1 |
|  | FEMALE | Count | 52 | 54 | 51 | 47 | 204 |
|  |  | % within Gender | 25.5 | 26.5 | 25.0 | 23.0 | 100.0 |
|  |  | % within Which of the hospitals do you patronize? | 57.1 | 60.7 | 55.4 | 50.5 | 55.9 |
|  |  | % of Total | 14.2 | 14.8 | 14.0 | 12.9 | 55.9 |
| Total | | Count | 91 | 89 | 92 | 93 | 365 |
|  |  | % within Gender | 24.9 | 24.4 | 25.2 | 25.5 | 100.0 |
|  |  | % within Which of the hospitals do you patronize? | 100.0 | 100.0 | 100.0 | 100.0 | 100.0 |
|  |  | % of Total | 24.9 | 24.4 | 25.2 | 25.5 | 100.0 |

Source: Researcher’s Field Survey, 2016

1. **The Respondents’ Gender:** Table 4.2.1a shows the frequency distribution of the gender of the respondents who are customers of the hospitals sampled. It is evident in Table 4.2.1a that male respondents constituted 161(44.1%) and females were 204 (55.9%) of the respondents. This result shows that there are more female respondents than the male in the surveys.

| **Table 4.2.1b : Age of Customer and Hospital Patronized** | | | | | | | |
| --- | --- | --- | --- | --- | --- | --- | --- |
|  | | | Hospitals Patronized | | | | Total n(%) |
|  |  |  | Lagoon Hospital | Reddington Hospital | EKO Hospital | St. Nicholas Hospital |  |
| Age | 18-30 | Count | 27 | 33 | 16 | 26 | 102 |
|  |  | Age | 26.5 | 32.4 | 15.7 | 25.5 | 100.0 |
|  |  | Hospital’s patronized | 29.7 | 37.1 | 17.4 | 28.0 | 27.9 |
|  |  | % of Total | 7.4 | 9.0 | 4.4 | 7.1 | 27.9 |
|  | 31-40 | Count | 59 | 34 | 47 | 36 | 176 |
|  |  | Age | 33.5 | 19.3 | 26.7 | 20.5 | 100.0 |
|  |  | Hospital’s patronized | 64.8 | 38.2 | 51.1 | 38.7 | 48.2 |
|  |  | % of Total | 16.2 | 9.3 | 12.9 | 9.9 | 48.2 |
|  | 41-50 | Count | 4 | 15 | 24 | 27 | 70 |
|  |  | Age | 5.7 | 21.4 | 34.3 | 38.6 | 100.0 |
|  |  | Hospital’s  Patronized | 4.4 | 16.9 | 26.1 | 29.0 | 19.2 |
|  |  | % of Total | 1.1 | 4.1 | 6.6 | 7.4 | 19.2 |
|  | 51-ABOVE | Count | 1 | 7 | 5 | 4 | 17 |
|  |  | Age | 5.9 | 41.2 | 29.4 | 23.5 | 100.0 |
|  |  | Hospital’s patronized | 1.1 | 7.9 | 5.4 | 4.3 | 4.7 |
|  |  | % of Total | 0.3 | 1.9 | 1.4 | 1.1 | 4.7 |
| Total | | Count | 91 | 89 | 92 | 93 | 365 |
|  |  | Age | 24.9 | 24.4 | 25.2 | 25.5 | 100.0 |
|  |  | Hospital’s patronized | 100.0 | 100.0 | 100.0 | 100.0 | 100.0 |
|  |  | % of Total | 24.9 | 24.4 | 25.2 | 25.5 | 100.0 |

Source: Researcher’s Field Survey, 2016

1. **Age Distribution of the Respondents:** Table 4.2.1b shows the distribution of the respondents by age. The result (Table 4.2.1b) reveals that 102 (27.9%) of the respondents were between the ages of 18years and 30years, 176(48.2%) were respondents between ages of 31years and 40years, 70 (19.2%) were respondents between ages of 41years and 50years and 17(4.7%) of the respondents were of the 51years and above. This result indicates that customers between the ages of 31years and 40years the hospitals patronized most. This is followed by those between 18years and 30years and those between 41years and 50years as well as those of 51years and above.

| **Table 4.2.1c: Highest Educational Qualification of the Customers and Hospital Patronized** |
| --- |

|  | Hospitals Patronized | | | |  |
| --- | --- | --- | --- | --- | --- |
| **WAEC O’ Level**  Educational Qualification  Hospital’s patronized?  % of Total  **B.SC.**  Highest Educational Qualification  Hospital’s patronized  % of Total  **MSC/MBA**  Educational Qualification  Hospital’s patronized  % of Total  **OTHERS**  Highest Educational Qualification  Hospital’s patronized  % of Total  Educational Qualification  Hospital’s patronized  % of Total | Lagoon Hospital | Reddington Hospital | EKO Hospital | St. Nicholas Hospital | Total n (%) |
|  | 11 | 10 | 7 | 11 | 39 |
|  | 28.2 | 25.6 | 17.9 | 28.2 | 100 |
|  | 12.1 | 11.2 | 7.6 | 11.8 | 10.7 |
|  | 3.0 | 2.7 | 1.9 | 3.0 | 10.7 |
|  | 75 | 62 | 39 | 60 | 236 |
|  | 31.8 | 26.3 | 16.5 | 25.4 | 100 |
|  | 82.4 | 69.7 | 42.4 | 64.5 | 64.7 |
|  | 20.5 | 17.0 | 10.7 | 16.4 | 64.7 |
|  | 4 | 12 | 31 | 20 | 67 |
|  | 6.0 | 17.9 | 46.3 | 29.9 | 100 |
|  | 4.4 | 13.5 | 33.7 | 21.5 | 18.4 |
|  | 1.1 | 3.3 | 8.5 | 5.5 | 18.4 |
|  | 1 | 5 | 15 | 2 | 23 |
|  | 4.3 | 21.7 | 65.2 | 8.7 | 100 |
|  | 1.1 | 5.6 | 16.3 | 2.2 | 6.3 |
|  | 0.3 | 1.4 | 4.1 | 0.5 | 6.3 |
|  | 91 | 89 | 92 | 93 | 365 |
|  | 24.9 | 24.4 | 25.2 | 25.5 | 100 |
|  | 100 | 100 | 100 | 100 | 100 |
|  | 24.9 | 24.4 | 25.2 | 25.5 | 100 |

Source: Researcher’s Field Survey, 2016

1. **Highest Educational Qualification of the Respondents:** Table 4.2.1c shows the highest educational qualification of the customers in the four hospitals sampled. It can be seen in Table 4.2.1c that 39(10.7%) of the respondents had WAEC, 236 (64.7%) had B.Sc, 67(18.4%) had M.Sc/MBA while the remaining 23(6.3%) had others educational qualifications apart from those listed in Table 4.2.1c. This result reveals that a majority of the respondents are well educated and able to provide the required information needed for this study. Further analysis of the result in Table 4.2.1c reveals that a majority of those who participated in the surveys as customers in the four hospitals had Bachelor degree as their highest educational qualification. This is followed by those with Master’s degree and those with WAEC O’ Level as their highest educational qualifications.

| **Table 4.2.1d: Marital Status of the Customers and Hospital Patronized** | | | | | | | |
| --- | --- | --- | --- | --- | --- | --- | --- |
|  | | | Hospitals Patronized | | | | Total n(%) |
|  |  |  | Lagoon Hospital | Reddington Hospital | EKO Hospital | St. Nicholas Hospital |  |
| Marital status | Single | Count | 33 | 30 | 19 | 28 | 110 |
|  |  | Marital status | 30.0 | 27.3 | 17.3 | 25.5 | 100.0 |
|  |  | Hospital’s patronized | 36.3 | 33.7 | 20.7 | 30.1 | 30.1 |
|  |  | % of Total | 9.0 | 8.2 | 5.2 | 7.7 | 30.1 |
|  | Married | Count | 57 | 57 | 72 | 63 | 249 |
|  |  | Marital status | 22.9 | 22.9 | 28.9 | 25.3 | 100.0 |
|  |  | Hospital’s patronized | 62.6 | 64.0 | 78.3 | 67.7 | 68.2 |
|  |  | % of Total | 15.6 | 15.6 | 19.7 | 17.3 | 68.2 |
|  | Divorced/separate | Count | 1 | 2 | 1 | 2 | 6 |
|  |  | Marital status | 16.7 | 33.3 | 16.7 | 33.3 | 100.0 |
|  |  | Hospitals patronized | 1.1 | 2.2 | 1.1 | 2.2 | 1.6 |
|  |  | % of Total | 0.3 | 0.5 | 0.3 | 0.5 | 1.6 |
| Total | | Count | 91 | 89 | 92 | 93 | 365 |
|  |  | Marital status | 24.9 | 24.4 | 25.2 | 25.5 | 100.0 |
|  |  | Hospital’s patronized | 100.0 | 100.0 | 100.0 | 100.0 | 100.0 |
|  |  | % of Total | 24.9 | 24.4 | 25.2 | 25.5 | 100.0 |

**Source: Researcher’s Field Survey, 2016**

1. **Marital Status of the Respondents:** The result in Table 4.2.1d shows the marital status of the respondents. This result reveals that 110 (30.1%) were single, 249 (68.2%) were married, while 6 (1.6%) were divorced/separated. This means that there are more married respondents that patronized the hospitals than the singles.

| **Table 4.2.1e: Occupation of the Customers and the Hospitals Patronized** | | | | | | | |
| --- | --- | --- | --- | --- | --- | --- | --- |
|  | | | Hospitals Patronized | | | | Total n(%) |
|  |  |  | Lagoon Hospital | Reddington Hospital | EKO Hospital | St. Nicholas Hospital |  |
| Occ upation | Student | Count | 43 | 22 | 3 | 27 | 95 |
|  |  | % within Occupation | 45.3 | 23.2 | 3.2 | 28.4 | 100.0 |
|  |  | % within Which of the hospitals do you patronize? | 47.3 | 24.7 | 3.3 | 29.0 | 26.0 |
|  |  | % of Total | 11.8 | 6.0 | 0.8 | 7.4 | 26.0 |
|  | Employe | Count | 8 | 17 | 22 | 20 | 67 |
|  |  | % within Occupation | 11.9 | 25.4 | 32.8 | 29.9 | 100.0 |
|  |  | % within Which of the hospitals do you patronize? | 8.8 | 19.1 | 23.9 | 21.5 | 18.4 |
|  |  | % of Total | 2.2 | 4.7 | 6.0 | 5.5 | 18.4 |
|  | Employee | Count | 40 | 50 | 67 | 46 | 203 |
|  |  | % within Occupation | 19.7 | 24.6 | 33.0 | 22.7 | 100.0 |
|  |  | % within Which of the hospitals do you patronize? | 44.0 | 56.2 | 72.8 | 49.5 | 55.6 |
|  |  | % of Total | 11.0 | 13.7 | 18.4 | 12.6 | 55.6 |
| Total | | Count | 91 | 89 | 92 | 93 | 365 |
|  |  | % within Occupation | 24.9 | 24.4 | 25.2 | 25.5 | 100.0 |
|  |  | % within Which of the hospitals do you patronize? | 100.0 | 100.0 | 100.0 | 100.0 | 100.0 |
|  |  | % of Total | 24.9 | 24.4 | 25.2 | 25.5 | 100.0 |

Source: Researcher’s Field Survey, 2016

1. **Occupation of the Customers:** The distribution of respondents according to their occupation shown in Table 4.2.1e. it is evident in Table 4.2.1e that 95 (26.0%) of the customers who participated in the survey were student, 67 (18.4%) were employers of labour, while 203(55.6%) were employees. This result suggests a majority of customer of the four private hospitals sampled are employees.

| **Table 4.2.1f : Type/class of customers and the hospitals patronized** | | | | | | | |
| --- | --- | --- | --- | --- | --- | --- | --- |
|  | | | Hospitals Patronized | | | | Total n(%) |
|  |  |  | Lagoon Hospital | Reddington Hospital | EKO Hospital | St. Nicholas Hospital |  |
| Respondent status with the firm | Corporate Customer | Count | 54 | 41 | 66 | 55 | 216 |
|  |  | Status with the firm | 25.0 | 19.0 | 30.6 | 25.5 | 100.0 |
|  |  | Hospital’s patronized? | 59.3 | 46.1 | 71.7 | 59.1 | 59.2 |
|  |  | % of Total | 14.8 | 11.2 | 18.1 | 15.1 | 59.2 |
|  | Private Individual | Count | 37 | 48 | 26 | 38 | 149 |
|  |  | Status with the firm | 24.8 | 32.2 | 17.4 | 25.5 | 100.0 |
|  |  | Hospital’s patronized? | 40.7 | 53.9 | 28.3 | 40.9 | 40.8 |
|  |  | % of Total | 10.1 | 13.2 | 7.1 | 10.4 | 40.8 |
| Total | | Count | 91 | 89 | 92 | 93 | 365 |
|  |  | % within Status with the firm | 24.9 | 24.4 | 25.2 | 25.5 | 100.0 |
|  |  | Hospital’s patronized? | 100.0 | 100.0 | 100.0 | 100.0 | 100.0 |
|  |  | % of Total | 24.9 | 24.4 | 25.2 | 25.5 | 100.0 |

Source: Researcher’s Field Survey, 2016

1. **Types/Classes of Customers:** Table 4.2.1f shows the different types or classes of customers of the hospitals that took part in the survey. It showed that 216(59.2%) were corporate customers, while the remaining 149 representing (40.8%) were private individuals. This result tends to suggest that more corporate than private customers patronized the four private hospitals investigated in this study.

| **Table 4.2.1g : Years of Patronage Experience and Hospital Patronized** | | | | | | | | | | | | |
| --- | --- | --- | --- | --- | --- | --- | --- | --- | --- | --- | --- | --- |
|  | | | | Hospitals Patronized | | | | | | | Total n(%) | |
|  |  |  |  | Lagoon Hospital | | Reddington Hospital | EKO Hospital | | | St. Nicholas Hospital |  |  |
| e Experience so far | 1 - 5 years | | Count | 51 | 62 | | | 64 | 43 | | | 220 |
|  |  |  | Patronage Experience | 23.2 | 28.2 | | | 29.1 | 19.5 | | | 100.0 |
|  |  |  | Hospital’s patronized | 56.0 | 69.7 | | | 69.6 | 46.2 | | | 60.3 |
|  |  |  | % of Total | 14.0 | 17.0 | | | 17.5 | 11.8 | | | 60.3 |
|  | 6 – 10 years | | Count | 27 | 19 | | | 19 | 41 | | | 106 |
|  |  |  | Patronage Experience | 25.5 | 17.9 | | | 17.9 | 38.7 | | | 100.0 |
|  |  |  | Hospital’s patronized | 29.7 | 21.3 | | | 20.7 | 44.1 | | | 29.0 |
|  |  |  | % of Total | 7.4 | 5.2 | | | 5.2 | 11.2 | | | 29.0 |
|  | 11 – 15 years | | Count | 8 | 6 | | | 3 | 5 | | | 22 |
|  |  |  | Patronage Experience | 36.4 | 27.3 | | | 13.6 | 22.7 | | | 100.0 |
|  |  |  | Hospital’s patronized | 8.8 | 6.7 | | | 3.3 | 5.4 | | | 6.0 |
|  |  |  | % of Total | 2.2 | 1.6 | | | 0.8 | 1.4 | | | 6.0 |
|  | 16 years and above | | Count | 5 | 2 | | | 6 | 4 | | | 17 |
|  |  |  | Patronage Experience | 29.4 | 11.8 | | | 35.3 | 23.5 | | | 100.0 |
|  |  |  | Hospital’spatronized | 5.5 | 2.2 | | | 6.5% | 4.3 | | | 4.7 |
|  |  |  | % of Total | 1.4 | 0.5 | | | 1.6 | 1.1 | | | 4.7 |
| Total | | Count | | 91 | | 89 | 92 | | | 93 | 365 | |
|  |  | Patronage Experience | | 24.9 | | 24.4 | 25.2 | | | 25.5 | 100.0 | |
|  |  | Hospital’s patronized | | 100.0 | | 100.0 | 100.0 | | | 100.0 | 100.0 | |
|  |  | % of Total | | 24.9 | | 24.4 | 25.2 | | | 25.5 | 100.0 | |

Source: Researcher’s Field Survey, 2016

1. **Years of Patronage Experience:** Table 4.2.1g is the presentation of the result on the customers’ years of patronage experience with the four hospitals. The result as presented in Table 4.2.1g reveals that 220 representing (60.3%) of the respondents had been patronizing the hospitals between the period of 1year and 5 years, 106(29.0%) had been using the hospitals for between 6years and 10years, 22 representing (6.0%) had patronized the hospitals for between 11years and 15years while 17(4.7%) of the respondents have been customers to the hospitals for 16 years and above. The result indicated that the majority of the respondent had been patronizing the hospital for at least five years and therefore qualified to provide reliable information for the current research.

## **4.2.2 Demographic Characteristics of the Healthcare Experts/Managers**

In the second phase of the research, data were collected from the healthcare managers. Out of the 160 copies of a questionnaire administered, 134 were returned. However, 124 were found usable and suitable for further analysis. This translates to 77.5% response rate.

**Table 4.2.2a: Response rate of questionnaires administered to the Healthcare Experts.**

| S/N | Hospitals | Num. Dist. | Number  Retrieved | Num. not Retrieved | Num. Fully Completed | Percent fully completed | Uncompleted |
| --- | --- | --- | --- | --- | --- | --- | --- |
| 1 | LAGOON HOSPITAL | 49 | 36 | 13 | 33 | 20.6 | 3 |
| 2 | REDDINGTON HOSPITAL | 36 | 30 | 6 | 27 | 16.9 | 3 |
| 3 | EKO HOSPITAL | 40 | 37 | 3 | 37 | 23.1 | 0 |
| 4 | ST. NICHOLAS HOSPITAL | 35 | 31 | 4 | 27 | 16.9 | 4 |
|  | TOTAL | 160 | 134 | 26 | 124 | 77.5 | 10 |

Source: Researcher’s Field Survey, 2016

**Table 4.2.2b** Sample Characteristics of Healthcare Experts/Managers

|  | LGH | RDTH | EKOH | STNH | Total |
| --- | --- | --- | --- | --- | --- |
| Number of target respondents | 49 | 36 | 40 | 35 | 160 |
| Share of actual respondents | 33 | 27 | 37 | 27 | 124 |
| Share of response rate **(%)** | 67.3 | 75.0 | 92.5 | 77.1 | 77.5 |
| Share of male **(in %)** | 21.2 | 88.9 | 44.6 | 59.5 | 56.5 |
| Share of female **(in %)** | 78.8 | 11.1 | 40.5 | 37.0 | 43.5 |
| Average age in years **(%)** | 64.8 | 38.2 | 51.1 | 38.7 | 42.2 |
| Share of respondents by patronage experience **(%)** | 56.0 | 69.7 | 69.6 | 46.2 | 60.3 |

Source: Researcher’s Field Survey, 2016

KEY: LGH = Lagoon Hospital; RDTH= Reddington Hospital,

EKOH = EKO Hospital; STNH = St. Nicholas Hospital

**Table 4.2.2c: Biographical Data of the Healthcare Experts/Managers**

| **Gender** | **Frequency** | **Percent** |
| --- | --- | --- |
| Male | 70 | 56.5 |
| Female | 54 | 43.5 |
| Total | 124 | 100.0 |
| **AGE** |  |  |
| 18-30 | 18 | 14.5 |
| 31-40 | 60 | 48.4 |
| 41-50 | 37 | 29.8 |
| 51 and above | 9 | 7.3 |
| Total | 124 | 100.0 |
| **Highest Educational Qualification** |  |  |
| B.Sc. | 48 | 38.7 |
| M.Sc | 32 | 25.8 |
| MBA | 10 | 8.1 |
| Others | 34 | 27.4 |
| Total | 124 | 100.0 |
| **Marital Status** |  |  |
| Single | 20 | 16.1 |
| Married | 102 | 82.3 |
| Divorced/separated | 2 | 1.6 |
| Total | 124 | 100.0 |
| **Name of the Organisation** |  |  |
| Lagoon Hospital | 33 | 26.6 |
| Reddington Hospital | 27 | 21.8 |
| EKO Hospital | 37 | 29.8 |
| St. Nicholas Hospital | 27 | 21.8 |
| Total | 124 | 100.0 |
| **Position in the Organisation** |  |  |
| Medical Doctors | 27 | 21.8 |
| Manager/Supervisor | 64 | 51.6 |
| Matron | 10 | 8.1 |
| Nurse | 16 | 12.9 |
| Others | 7 | 5.6 |
| Total | 124 | 100.0 |
| **Length of Service in the Organisation** |  |  |
| 1-5 years | 62 | 50.0 |
| 6-10 | 45 | 36.3 |
| 11-15 | 7 | 5.6 |
| 16 years and above | 10 | 8.1 |
| Total | 124 | 100.0 |

Source: Researcher’s Field Survey, 2016

1. **Gender:** From the data in Table 4.2.2c, the following can be seen. The distribution of the healthcare experts and managers shows that 70(56.5%) of the respondents were male, while 54 (43.5%) were female. This result suggests that there are more males than female healthcare expert/managers in these four private hospitals
2. **Age:** Table 4.2.2c also shows distribution of the healthcare experts/managers according to their age. The result (Table 4.2.2c) shows that 18 (14.5%) of the healthcare experts/manager sampled were between 18years and 30years of ages, 60(48.4%) were between the ages of 31years and 40 years, 37(29.8%) were between 41 years and 50 years, while 9(7.3%) of them were 51years and above. This suggests that a high majority of the healthcare experts that participated in the research are between 31years and 40 years of age.
3. **Highest Educational Qualification:** The highest educational qualifications of the healthcare experts as displayed in Table 4.2.2c that 48(38.7%) had B.Sc, 23(25.8) had M.Sc, 10(8.1%) had MBA while the remaining 34(27.4) had others educational qualifications. This shows that most of the healthcare experts/managers in the four hospitals are degree holders

1. **Marital Status:** Table 4.2.2c also shows the marital status of the respondents who are healthcare experts. It is evident from the Table that 20 (16.1%) were single, 102(82.3%) were married and 2(1.6%) of them were divorced/separated. This means that there are more married respondents among the healthcare experts of the hospitals than the singles. Therefore, a high majority of the healthcare experts/managers were family men and women.
2. **Name of the organisation:** The Table 4.2.2c shows the distribution of the healthcare experts by the hospital name. It is evident that 33(26.6%) were staff of Lagoon Hospital, 27 representing 21.8% were staff of Reddington and St. Nicholas Hospital and the remaining 37 representing (29.8%) were staff of EKO Hospital. This means that there are more respondents from EKO hospital than the other hospitals.
3. **Role in the organisation:** The data in Table 4.2.2c reveal the different roles of the staff in the hospitals sampled. It can be seen from the result also that 27(21.8%) of them were Medical Doctors, 64 representing 51.6% were managers/supervisors, 10 representing 8.1% were Matron of the hospitals 16 representing 12.9 were nurses and the remaining 7 representing 5.6% indicated other categories of staff. This means that the managers/supervisors in the hospital had the highest number of the respondents in the survey, and the key healthcare services providers in the hospitals were sampled.
4. **Length of Service in the Organisation:** From Table 4.2.2c it is also evident that 62(50.0%), of staff sampled have been working with the hospitals for between 1year and 5 years, 45 representing 36.3% of the respondents have been working with the hospital for between 6years and 10 years, 7 representing 5.6% of the respondents have been working with the hospital for between 11years and 15 years, while 10 representing the 8.1% of them indicate that they had worked in the hospital for 16year and above. This means that a majority of the sampled respondents have been working with the at least 5years, and therefore considered qualified to provide information on customer experience management in their respective hospitals.

## **4.2.3 Healthcare Experts’ Perception of the Objectives of the Study**

| **Components of Functional Clues** | **N** | **Sum** | **RII** | **Rank** |
| --- | --- | --- | --- | --- |
| The competence of our health care team | 124 | 560 | 0.903 | 1 |
| The quality of our health care services | 124 | 558 | 0.900 | 2 |
| The reliability of our health care facilities | 124 | 553 | 0.892 | 3 |
| The efficacy of our health care product | 124 | 545 | 0.879 | 4 |
| The commitment of our health care team to right diagnosis | 124 | 544 | 0.877 | 5 |
| The efficacy of the drugs dispensed to patients | 124 | 526 | 0.848 | 6 |
| Valid N (listwise) | 124 |  |  |  |

**TABLE 4.2.3a: The Role of Functional Clues on Repeat Purchase**

Source: Researcher’s Field Survey, 2016

Table 4.2.3a shows result of the descriptive statistics of the roles of functional clues on repeat purchase based on the views of the healthcare experts. Using the relative important index or RII formula, The Relative Importance Index (RII) for each of the variables used to investigate the role of functional clues on repeat purchase was computed.

RII= (**Σ**^5^i_=1_ W_i_ X f_xi_)

N (A)

where,

w = The weight age of the respondents

A= 1, 2, 3, 4, 5

f_xi_ = The frequency of every respondent

N = Total number of respondents (Hamzah, Khoiry, Ali, Zaini & Arshal, 2011, Tawil, Hamzah, Khoiry, Ani & Basri, 2011).

According to several scholars, the standard deviations and mean are not dependable measurements for weighing overall ranking of the elements (Doloi, 2008). RII provides a descriptive interpretation of the most important element (Doloi and Young, 2009). Therefore, in this study, the RII was considered appropriate in examining which components of the functional clues are ranked highest in eliciting repeat purchase of healthcare services among the customers of the four private hospitals investigated.

Functional clue in this context refers to the reliability and competence of the services provided by the health care organisations. The RII gotten from the formula was employed to determine the highest score of all the responses listed. The highest ranked for each of the responses has been decided based on the opinions of the respondents. Going by the result, as shown in Table 4.2.3a the competence of the healthcare team has the highest RII, followed by the quality of healthcare services and the reliability of healthcare facilities. This means that the healthcare expert in the hospitals sampled agreed or strongly agreed that the competence of the healthcare team, the quality of healthcare services and the reliability of healthcare facilities contribute most to customers’ willingness to patronize the organisation again. However, looking at the RII in Table 4.2.3a, it is evident that there is a slight difference between the efficacy of the healthcare product and the commitment of the healthcare team with the RII value of (0.879 and 0.877). Also, based on this result, it can be inferred that the respondents positively agreed or strongly agreed with all the variables concerning the roles of functional clues on repeat purchase actions. It is however important to note that the competence of the healthcare team appears to contribute most to repeat purchase actions by the customers of the health institutions investigated in this research.

**TABLE 4.2.3b: Healthcare Experts’ Perception of the Effect of Mechanic Clues on Brand Insistence**

| **Components of Mechanic Clues** | **N** | **Sum** | **RII** | **Rank** |
| --- | --- | --- | --- | --- |
| The cleanliness of our health care offices | 124 | 546 | 0.881 | 1 |
| The adequacy of our health care facilities | 124 | 531 | 0.856 | 2 |
| The suitability of the location of our health care institutions | 124 | 525 | 0.847 | 3 |
| The conducive ambience of our health care organisation | 124 | 504 | 0.813 | 4 |
| The lighting system of health care organisation | 124 | 486 | 0.784 | 5 |
| The physical esthetics of our health care facility | 124 | 485 | 0.782 | 6 |
| The furniture and fitting of health care facility | 124 | 481 | 0.776 | 7 |

Source: Researcher’s Field Survey, 2016

Table 4.2.3b shows the descriptive statistics of the influence of mechanic clues on brand insistence using the relative important index. The data in Table 4.2.3b was designed to find out healthcare experts’ view about the influence of physical environments such as furnishings, building design, displays, equipment, colours, smells, sounds, lighting and the appearance of the health care organisation on brand insistence. From result, four of seven components, namely: The cleanliness of the healthcare offices, the adequacy of the healthcare facilities, the suitability of the location of the institution and the conducive ambience of the health care organisation were establish to be amongst the major contributors to brand insistence in this study. This means that the respondents of the sampled hospital agreed that the cleanliness of the healthcare organisation, the adequacy of the healthcare facilities, the suitability of the location of health care institutions and the conducive ambience of our health care organisation influenced customers’ patronage. Specifically, all other statement related to the mechanic clues also revealed positive response but with a slight difference in the RII value of the other items (0.784, 0.782 and 0.776). This is due to the fact that the respondents agreed to the statements on mechanic clues. Based on these results, it is evident that the respondents agreed or strongly agreed with all the statements concerning the mechanic clues and that the cleanliness of the environment of the healthcare institutions contribute most to brand insistence by customers in the healthcare sector.

**TABLE 4.2.3c: Healthcare Experts’ Perception of Humanic Clues on Switching Restraint**

| **Components of Humanic Clues** | **N** | **Sum** | **RII** | **Rank** |
| --- | --- | --- | --- | --- |
| The health care service providers show respect and courtesy to their patients (customers) | 124 | 532 | 0.858 | 1 |
| Friendliness of the health care service providers to their customers | 124 | 523 | 0.844 | 2 |
| The behaviour of the health care service provider is consistently positive towards the patients (customers). | 124 | 518 | 0.835 | 3 |
| The health care service providers are consistently caring | 124 | 516 | 0.832 | 4 |
| The tone of voice of the health care service providers are pleasant | 124 | 503 | 0.811 | 5 |
| The body language of the health care service providers is encouraging to the patients (customers). | 124 | 480 | 0.774 | 6 |

Source: Researcher’s Field Survey, 2016

The Table 4.2.3c shows result of the descriptive statistics of the influence of humanic clues on customer’s switching restraint using the relative important index. It can be seen from Table 4.2.3c that respect and courtesy to the customer had the highest RII value of 0.858, followed by the friendliness of the healthcare service provider with the RII value of 0.844, while the caring behaviour of the healthcare service provider and their consistent caring have RII value with slight difference of 0.835 and 0.832. In addition, the tone of voice of the healthcare service providers with RII value of 0.811and lastly the body language of the healthcare service providers has RII value of 0.774. This means that the respondents’ agreed that the respect and courtesy shown by the health care service providers has the strongest influence on switching restraints. From this result, it is clear that the respondents in the survey agreed with all the statements concerning the influence of humanic clues on switching restraint. Therefore, it can be inferred that humanic clues influence the switching restraint of customers in the four private hospitals sampled.

**4.2.4: CUSTOMERS’ PERCEPTION OF THE OBJECTIVES OF THE STUDY**

**Table 4.2.4a: The Role of Functional Clues on Repeat Purchase**

| **Component of Functional Clues** | **Total Score** | **RII** | **Rank** |
| --- | --- | --- | --- |
| The competence of the health care service provider | 1513 | 0.829 | 1 |
| The right diagnosis by the health care organisation. | 1486 | 0.816 | 2 |
| Administration of the right treatment by the health care organisation | 1478 | 0.810 | 3 |
| The reliability of the health care services | 1473 | 0.807 | 4 |
| The assured service of the health care organisation | 1473 | 0.807 | 4 |
| The reliability of the services by the healthcare institutions. | 1464 | 0.802 | 5 |
| The self-reliance of the health care service | 1450 | 0.795 | 6 |
| The efficacy of the health care services | 1447 | 0.793 | 7 |
| The practicality of the healthcare services | 1417 | 0.776 | 8 |
| The procedures of the health care services | 1403 | 0.769 | 9 |

Source: Researcher’s Field Survey, 2016

Table 4.2.4a shows the descriptive statistics of the role of functional clues on repeat purchase actions based on the customers’ perspective using the relative important index. Functional clue in this context refers to the reliability and competence of the services provided by the health care organisation. This aspect of the research was designed to measure the customers’ perspective opinion on the role of functional clues on their repeat purchase actions. From the result (Table 4.2.4a) it is evident that the competence of the health care service providers has the highest RII value of 0.829 and ranked as the first. The right diagnosis is ranked the second with the RII value of 0.816, while the administration of the right treatment is ranked the third with the RII value of 0.810. Meanwhile, both the reliability of the healthcare service and the assured services of the healthcare services have equal RII value of 0.807. In addition, Table 4.2.4a also reveals that there is a slight difference among the RII values of the remaining four items (0.795, 0.793, 0.776 and 0.769). Further, a critical review of the RII values in Table 4.2.4a will show all the statements on the functional clues play important roles in eliciting repeat purchase actions of customers in the healthcare sector. Generally, the result is an indication that the respondents agreed with all the statements concerning the roles functional clues on the repeat purchase actions.

**Table 4.2.4b: The Influence of Mechanic Clues on Brand Insistence**

| **Components of Mechanic Clues** | **Total Score** | **RII** | **Rank** |
| --- | --- | --- | --- |
| The cleanliness of the health care organisation | 1550 | 0.849 | 1 |
| The physical appearance of the health care organisation | 1479 | 0.810 | 2 |
| The facilities of the health care organisation | 1478 | 0.809 | 3 |
| The comfort of the health care lobby/reception area | 1465 | 0.803 | 4 |
| The lighting of the health care organisation | 1454 | 0.797 | 5 |
| The conducive ambience of the health care organisation | 1449 | 0.794 | 6 |
| The convenient location of the health care organisation | 1444 | 0.791 | 7 |
| The furniture and fitting of the health care organisation | 1332 | 0.730 | 8 |
| The aroma of the health care organisation | 1265 | 0.693 | 9 |

Source: Researcher’s Field Survey, 2016

Table 4.2.4b shows result of the descriptive statistics of the influence of mechanic clues, that is the physical environments such as furnishings, building design, displays, equipment, colours, smells, sounds, lighting and the appearance of the health care facilities on brand insistence using the relative important index. The result reveals that the cleanliness of the healthcare organisation has the highest RII value of 0.849, followed by the physical appearance and the facilities of the healthcare organisation, which emerged with RII value of 0.810 and 0.809 respectively. The comfort of the healthcare lobby/reception has the RII value of 0.803. This means that the respondents in the hospitals sampled strongly agreed that the cleanliness of the healthcare organisation, the physical appearance, the facilities of the healthcare organisation and the comfort of the healthcare lobby/reception contribute positively in making the customers insist on the services of the organisation, while all other statements related to the mechanic clues on brand insistence also revealed positive responses but with a slight difference among the RII value of the remaining items. Given these results, it is obvious that the respondents agreed or strongly agreed with all the statements concerning the influence of mechanic clues on brand insistence.

**Table 4.2.4c: The Effects of Humanic Clues on Switching Restraints**

| **Components of Humanic Clues** | **N** | **Sum** | **RII** | **Rank** |
| --- | --- | --- | --- | --- |
| The friendly actions of the health care service providers | 364 | 1509 | 0.829 | 1 |
| The neatness of the health care service providers | 365 | 1512 | 0.828 | 2 |
| The respect and courtesy from the health care service providers | 365 | 1488 | 0.815 | 3 |
| The caring expression of the health care service providers | 365 | 1486 | 0.814 | 4 |
| The understanding of the health care service providers | 365 | 1480 | 0.811 | 5 |
| The mindfulness of the health care service providers | 365 | 1455 | 0.797 | 6 |
| The tone of voice of the health care service providers | 364 | 1449 | 0.796 | 7 |
| The responsiveness of the health care service providers | 365 | 1419 | 0.778 | 8 |
| The body language of the health care service providers | 365 | 1367 | 0.749 | 9 |

Source: Researcher’s Field Survey, 2016

The data in Table 4.2.4c shows the descriptive statistics of the effects of humanic clues on switching restraint. This aspect of the research was designed to examine the customers’ views on the influence of the actions, behaviour and expressions of the employees of the healthcare organisation on switching restraint using the relative important index. The RII values of the respondents on whether the actions, behaviour and expressions of the employees of the healthcare organisations make them continually patronize the organisations revealed 0.829, 0.828, 0.815, 0.814 and 0.811 for the different components of humanic clues. This result tends to show that the respondents of the hospitals sampled agreed that the friendly actions, neatness, respect and courtesy, expressions and the understanding of the employees of the healthcare organisations make them continually restrain switching to the healthcare organisations. Similarly, other statements related to the humanic clues namely: the mindfulness of the health care service provider, the tone of voice of the health care service providers, the responsiveness of the health care service providers and the body language of the health care service providers also revealed positive response but with a slight difference among the RII value of the remaining items. This is due to the fact that the sampled respondents agreed with the stateme\\nts on the effects of humanic clues on switching restraint. Generally speaking, the result is an indication that the respondents agreed or strongly agreed with all the statements concerning the effects of humanic clues on switching restraint.

**Table 4.2.4d: The Relationship between Customer Experience and Customer Satisfaction**

| **Components of Customer Experience and Customer Satisfaction** | **N** | **Mean(Satisfaction score)** | **Std. Deviation** | **Rank** |
| --- | --- | --- | --- | --- |
|  | **Statistic** | **Statistic** | **Statistic** |  |
| Showing professionally appropriate behaviour | 365 | 4.17 | .825 | 1 |
| Maintaining patient privacy | 365 | 4.17 | .881 | 1 |
| Right treatment of illness | 365 | 4.16 | .771 | 2 |
| Reliability of the health care services. | 365 | 4.12 | .838 | 3 |
| Cleanliness of the health care organisation | 365 | 4.10 | .881 | 4 |
| Right diagnosis of illness | 365 | 4.08 | .806 | 5 |
| Effective verbal communication. | 365 | 4.06 | .864 | 6 |
| Respecting my wishes | 365 | 3.96 | .953 | 7 |
| Aroma of the health care organisation | 365 | 3.75 | 1.150 | 8 |
| Non-verbal communication. | 365 | 3.66 | 1.074 | 9 |
| Valid N (listwise) | 365 |  |  |  |

Source: Researcher’s Field Survey, 2016

Table 4.2.4d shows result of the analysis on customer experience and customer satisfaction. On a 5-linkert scale, the mean score of the respondents on showing professionally appropriate behaviour and maintaining their privacy revealed equal means score of 4.17 which is the highest. This suggests that the respondents in the hospitals sampled are most satisfied with professionally appropriate behaviour and maintaining their privacy. These are followed by the right treatment of illness which revealed mean scores of 4.16, reliability of the health care services with a mean scores of 4.12, cleanliness of the health care organisation with mean scores of 4.10, the right diagnosis of illness (4.08), and the effective verbal communication (4.06). The result revealed higher mean scores at different degrees compared to the other statement like respecting my wishes which showed mean scores of 3.96, while, the aroma of the health care organisation had mean score of 3.75 and lastly, the non-verbal communication, which had mean score of 3.66. This means that the respondents sampled in the hospitals have a positive response to all the statements concerning the relationship between customer experience and customer satisfaction. A further examination of the mean scores in Table 4.2.4d will show that no variable mean score is less than 3.01 on a 5-linkert point scale. This is an indication that the respondents agreed or strongly agreed that customer experience has a significant relationship with customer satisfaction.

**Table 4.2.4e: Buyer’s Psychological Factors on the Relationship between Customer Experience Management and Loyalty**

| **Components of Psychological factors, CEM and Loyalty** | **N** | **Mean** | **Std. Deviation** | **Rank** |
| --- | --- | --- | --- | --- |
|  | **Statistic** | **Statistic** | **Statistic** |  |
| My perception about the reliability of services. | 365 | 4.17 | .846 | 1 |
| My personality determines how I view the health care services. | 364 | 4.13 | .818 | 2 |
| My motivation about the quality of health care | 365 | 4.08 | .831 | 3 |
| My knowledge about the cleanliness of heath care | 365 | 4.07 | .804 | 4 |
| My interpretation of the right diagnosis of illness. | 365 | 4.06 | .818 | 5 |
| My experience with the behaviour of the health care service provider. | 365 | 4.04 | .862 | 6 |
| The belief I have concerning the right treatment of illness. | 365 | 4.03 | .916 | 7 |
| My view about the effective verbal communication | 365 | 4.01 | .879 | 8 |
| The maintenance of patient privacy. | 365 | 4.00 | .885 | 9 |
| My interpretation of the tone of voice of the health care service provider. | 365 | 3.86 | 1.000 | 10 |
| The aroma/odor of the hospital. | 365 | 3.68 | 1.112 | 11 |
| Valid N (listwise) | 364 |  |  |  |

Source: Researcher’s Field Survey, 2016

The data in Table 4.2.4e shows the descriptive statistics of the respondents’ opinion on whether their perception, motivation, learning and belief & attitudes (i.e. the psychological factors) moderate the relationship between their experience with the organisation and loyalty. The result (Table 4.2.4e) reveals positive responses concerning all the statements related to buyers’ psychological factors as seen in the mean scores, which are more than 3.01 on a 5-likert type scale. This table revealed 4.17, 4.13, 4.08, 4.07, 4.06, 4.04, 4.03, 4.01, 4.00, 3.86 and 3.68 respectively. Given the totality of the above result, it is an indication that the respondents agreed or strongly agreed that buyers’ psychological factors moderate the relationship between customer experience and customer loyalty.

##

## **4.2.5 CONSUMER’S PERCEPTION OF RESEARCH VARIABLES BASED ON DEMOGRAPHIC CHARACTERISTICS**

**Descriptive analysis of research objectives based on the gender of the respondents**

This section deals with the descriptive analysis of the research objectives based on respondents’ perception of the roles of functional clues on repeat purchase. These analyses were executed with the aim of examining whether there exists any difference in respondents perception of the roles of functional clues on repeat purchase based on the demographic characteristics of the customers.

**TABLE 4.2.5a : Roles of Functional Clues on Repeat Purchase**

| **Components of Functional Clues and Repeat Purchase** | **Mean Scores for Males** | **Mean Scores for Females** |
| --- | --- | --- |
| The reliability of the health care services. | 3.97 | 4.09 |
| The competence of the health care service. | 4.02 | 4.24 |
| The right diagnosis of the health care organisation. | 4.01 | 4.14 |
| Administration of the right treatment by the health care organisation. | 3.92 | 4.15 |
| The assured service of the health care organisation. | 3.92 | 4.13 |
| The procedures of the health care services. | 3.65 | 4.00 |
| The reliability of the continuous services. | 3.91 | 4.09 |
| The efficacy of the health care service. | 3.89 | 4.02 |
| The self-reliance of the health care service. | 3.91 | 4.02 |
| The practicality of the healthcare services. | 3.81 | 3.94 |

Source: Researcher’s Field Survey, 2016

| Table 4.2.5a shows the mean score of respondents’ rating of the roles of functional clues on repeat purchase by gender. This descriptive analysis was executed to assess whether there is any difference in respondents rating on the roles of functional clues on repeat purchase between male and female customers. From Table 4.2.5a, it is obvious that both male and female respondent share similar views on the roles of functional clues on repeat purchase. The result in fact also reveals positive responses on all the statements related to the roles of functional clues in eliciting repeat purchase as all the variables mean scores investigated have more than the 3.01 on a 5-Likert point scale. These findings point out that both male and female customers are likely to make a repeat purchase as a result of the reliability and the competence of the healthcare service delivery. The result also indicates that there are no significant differences in the role functional clues play in ensuring repeat purchase behaviour of male and female customers. Both male and female customers are likely to react similarly to functional clues when it comes to healthcare services. |
| --- |

**TABLE 4.2.5b: The Influence of Mechanic Clues on Brand Insistence**

| **Components of Mechanic Clues and Brand Insistence** | **Mean Scores for Males** | **Mean Scores for Females** |
| --- | --- | --- |
| The physical appearance of the health care organisation. | 4.06 | 4.04 |
| The conducive ambience of the health care organisation. | 4.06 | 3.90 |
| The furniture and fitting of the health care organisation. | 3.65 | 3.65 |
| The facilities of the health care organisation. | 4.02 | 4.07 |
| The lighting of the health care organisation. | 3.86 | 4.08 |
| The cleanliness of the health care organisation. | 4.17 | 4.31 |
| The comfort of the health care lobby/reception area. | 3.99 | 4.03 |
| The convenient location of the health care organisation. | 3.92 | 3.99 |
| The aroma of the health care organisation. | 3.45 | 3.48 |

| Source: Researcher’s Field Survey, 2016  The data in Table 4.2.5b is the result of the respondents’ opinion on the influence on mechanic clues on brand insistence across both sexes. From the result in Table 4.2.5b, it is evident that both the male and female customers have similar views on the influence of mechanic clues on brand insistence. This is because the result reveals positive responses on all the statements related to the effect of mechanic clues on brand insistence as all the mean scores are more than the 3.01 on a 5-linkert point scale. These findings indicate that both genders are likely to insist on brand as a result of the physical environments such as furnishing, building design, displays, equipment, colours, smells, sounds, lighting and the appearance of the health care facilities. Based on the result, it can be inferred that there are no gender differences in the perceptions of the influence of mechanic clues on brand insistence. This suggests that both male and female customers are likely to respond in the same way to mechanic clues associated with healthcare services. |
| --- |

**TABLE 4.2.5c: The Influence of Humanic Clues on Switching Restraint**

| **Components of Mechanic Clues and Switching Restraint** | **Mean Scores for Males** | **Mean Scores for Females** |
| --- | --- | --- |
| The friendly actions of the health care service providers. | 4.15 | 4.14 |
| The caring expression of the health care service providers. | 4.03 | 4.10 |
| The tone of voice of the health care service provider pleases me. | 3.82 | 4.11 |
| The body language of the health care service provider. | 3.75 | 3.75 |
| The respect and courtesy from the health care service provider. | 4.13 | 4.03 |
| The neatness of the health care service provider fascinates me. | 4.09 | 4.19 |
| The responsiveness of the health care service provider. | 3.85 | 3.92 |
| The mindfulness of the health care service provider. | 3.94 | 4.02 |
| The understanding of the health care service provider. | 4.10 | 4.02 |

| Source: Researcher’s Field Survey, 2016    The result in Table 4.2.5c shows the responses by the male and female customers on how humanic clues influence switching restraint. From the result, it is evident that both the male and female customers have similar responses on the influence of humanic clues on switching restraint. The result revealed positive responses concerning all the statements related to the influence of humanic clues on switching restraint as all the mean scores of the variables investigated are more than the 3.01 on a 5-likert type scale. These findings indicate that both genders are likely to be loyal to healthcare organisations as a result of the actions, behaviour and expressions of their employees. The findings presented in Table 4.2.5c also suggest that there are no gender differences in the perception of the influence of humanic clues on switching restraint as both male and female are likely to respond similarly to the influence of humanic clues when it comes to switching restraint. |
| --- |

**TABLE 4.2.5d: The Relationship between Customer Experience and Customer**

**Satisfaction**

| **Components of Customer Experience and satisfaction with healthcare services** | **Mean Scores for Males** | **Mean Scores for Females** |
| --- | --- | --- |
| The reliability of the health care services makes me contented. | 4.17 | 4.08 |
| The right diagnosis of illness by the health care organisation makes me completely happy with the organisation. | 3.99 | 4.15 |
| The right treatment of illness by the health care organisation enhances my relief. | 4.17 | 4.16 |
| The effective verbal communication of the health care service enhances my gratification. | 3.98 | 4.12 |
| The non-verbal communication of the health care service enhances my gratification. | 3.47 | 3.81 |
| Respecting my wishes makes me satisfied. | 3.87 | 4.04 |
| Showing professionally appropriate behaviour by the health care service provider delights me | 4.12 | 4.21 |
| Maintaining patient privacy enhances customer’s trust. | 4.14 | 4.19 |
| The aroma of the health care organisation makes me satisfied | 3.64 | 3.84 |
| The cleanliness of the health care organisation satisfies me | 4.11 | 4.09 |

| Source: Researcher’s Field Survey, 2016    The result of respondents’ perception of the relationship between their experience and satisfaction with healthcare services in the four hospitals is presented in Table 4.2.5d. From the result, it is obvious that both the male and female customers have similar views on the relationship between their experience and satisfaction. The Table also revealed positive responses concerning all the statements related to the relationship between customer experience and customer satisfaction as all the mean scores are more than the 3.01 on a 5-likert type scale. These findings signify that both male and female customers are likely to be satisfied with the services of healthcare organisations as a result of customer experience management strategies engaged in by the organisations. Further, findings in Table 4.2.5d also reveal that there are no gender differences in the perception of the relationship between customer experience and customer satisfaction with healthcare services. As both male and female are likely to respond in the same way to the relationship between customer experience and customer satisfaction. |
| --- |

**TABLE 4.2.5e: How Buyers’ Psychological Factors Moderate the Relationship between CEM & Loyalty.**

| **Components of Psychological factors, CEM and Loyalty** | **Mean Scores for Males** | **Mean Scores for Females** |
| --- | --- | --- |
| My perception about the reliability of services provided | 4.22 | 4.12 |
| My interpretation of the right diagnosis of illness. | 4.05 | 4.07 |
| My motivation about the quality of health care services. | 4.10 | 4.07 |
| My experience with the behaviour of the health care service provider. | 4.08 | 4.01 |
| The belief I have concerning the right treatment of illness provided by the organisation. | 3.96 | 4.09 |
| The maintenance of patient privacy by the healthcare organisation. | 3.96 | 4.03 |
| The aroma/odor of the healthcare organisation. | 3.60 | 3.74 |
| My view about the effective verbal communication of the healthcare service provider. | 4.00 | 4.02 |
| My knowledge about the cleanliness of heath care organisation surroundings. | 4.04 | 4.09 |
| My interpretation of the tone of voice of the health care service provider. | 3.77 | 3.93 |
| My personality determines how I view the health care services provided. | 4.14 | 4.12 |

Source: Researcher’s Field Survey, 2016

Regarding the perception of how the customers’ psychological factors moderate the relationship between customer experience management and customer loyalty, the result in Table 4.2.5e reveals that both the male and female customers have similar response pattern on this. Specifically, the results indicate positive responses concerning all the statements related to how buyers’ psychological factors moderate the relationship between customer experience management and customer loyalty. This Table revealed that the perception of customer about the reliability of services provided contributes most in moderating the relationship between customer experience management and customer loyalty as it reveals the highest mean scores of 4.22 for male and female 4.12, meanwhile the female are of the opinion that both perception and personality contribute most as it gives the mean scores of 4.12. The findings show that the gender of the respondents makes no significant difference on how buyers’ psychological factors moderate the relationship between customer experience management and customer loyalty when it comes to healthcare services delivered by the four private hospitals investigated.

## **4.2.6 CUSTOMERS’ PERCEPTION OF RESEARCH OBJECTIVES BASED ON AGE OF THE RESPONDENTS**

**TABLE 4.2.6a: Roles of functional clues on repeat purchase**

| **S/N** | **Components of Functional Clues and Repeat Purchase** | **(18-30)yrs** | **(31-40)yrs** | **(41-50)yrs** | **(51yrs +)** |
| --- | --- | --- | --- | --- | --- |
|  |  | **Mean scores** | **Mean scores** | **Mean scores** | **Mean**  **scores** |
| 1 | The reliability of the health care services. | 3.96 | 3.99 | 4.26 | 4.06 |
| 2 | The competence of the health care service. | 4.19 | 4.10 | 4.19 | 4.24 |
| 3 | The right diagnosis of the health care organisation. | 4.06 | 4.10 | 4.04 | 4.24 |
| 4 | Administration of the right treatment by the health care organisation. | 3.99 | 3.99 | 4.21 | 4.35 |
| 5 | The assured service of the health care organisation. | 3.94 | 4.02 | 4.17 | 4.18 |
| 6 | The procedures of the health care services. | 3.74 | 3.84 | 3.99 | 4.00 |
| 7 | The reliability of the continuous services. | 3.97 | 4.01 | 4.04 | 4.18 |
| 8 | The efficacy of the health care service. | 3.97 | 3.93 | 4.00 | 4.12 |
| 9 | The self-reliance of the health care service organisation. | 4.06 | 3.90 | 4.01 | 4.06 |
| 10 | The practicality of the healthcare services. | 3.89 | 3.80 | 4.01 | 4.18 |

Source: Field Survey, 2016

Based on the result in Table 4.2.6a, it is clear that the customers’ responses on the role of functional clues on repeat purchase actions across the different age of the respondents are positive on all the statements as all the mean scores are more than the 3.01 on a 5-Likert type scale. The findings show that age of the respondents makes no significant difference on their perception of the role of functional clues on repeat purchase when it comes to healthcare services provided by the four private hospitals investigated.

| **S/N** | **Components of mechanic Clues and Brand Insistence** | **18-30**  **years** | **31-40**  **years** | **41-50**  **years** | **51 years +** |
| --- | --- | --- | --- | --- | --- |
|  |  | **Mean scores** | **Mean scores** | **Mean scores** | **Mean scores** |
| 1 | The physical appearance of the health care organisation | 3.95 | 4.09 | 4.16 | 3.82 |
| 2 | The conducive ambience of the health care organisation | 4.00 | 3.93 | 4.01 | 4.00 |
| 3 | The furniture and fitting of the health care organisation | 3.53 | 3.60 | 3.94 | 3.71 |
| 4 | The facilities of the health care organisation. | 4.06 | 4.05 | 4.00 | 4.24 |
| 5 | The lighting of the health care organisation. | 4.03 | 3.95 | 3.94 | 4.18 |
| 6 | The cleanliness of the health care organisation. | 4.20 | 4.29 | 4.23 | 4.18 |
| 7 | The comfort of the health care lobby/reception area. | 3.96 | 3.96 | 4.21 | 4.06 |
| 8 | The convenient location of the health care organisation. | 3.91 | 3.93 | 4.11 | 3.88 |
| 9 | The aroma of the health care organisation | 3.16 | 3.53 | 3.74 | 3.53 |

**TABLE 4.2.6b: Customers’ perception of the influence of mechanic clues on brand**

**insistence**

Source: Field Survey, 2016

The data in Table 4.2.6b show respondents’ opinion on the influence on mechanic clues on brand insistence across the four age groupings. From the result in Table 4.2.6b, it is evident that all the age groups of the respondents have similar response pattern on the influence of mechanic clues on brand insistence. The result also reveals positive responses concerning all the statements related to the influence of mechanic clues on brand insistence as the mean scores for each of the nine items are more than the 3.01 on a 5-Likert type scale. These findings indicate that the respondents in all age group are likely to insist on a brand as a result of the physical environments such as furnishings, building design, displays, equipment, colours, smells, sounds, lighting and the appearance of the health care facilities of the organisations.

**TABLE 4.2.6c: Influence of humanic clues on switching restraint**

| S/N | **Components of Mechanic Clues and Brand Insistence** | **18-30**  **years** | **31-40**  **years** | **41-50**  **years** | **51 years+** |
| --- | --- | --- | --- | --- | --- |
|  |  | **Mean scores** | **Mean scores** | **Mean scores** | **Mean scores** |
| 1 | The friendly actions of the health care service providers. | 4.16 | 4.03 | 4.39 | 4.24 |
| 2 | The caring expression of the health care service providers. | 4.10 | 4.02 | 4.10 | 4.35 |
| 3 | The tone of voice of the health care service provider pleases me. | 3.70 | 4.10 | 4.04 | 4.12 |
| 4 | The body language of the health care service provider. | 3.77 | 3.69 | 3.83 | 3.82 |
| 5 | The respect and courtesy from the health care service provider. | 4.00 | 4.12 | 4.01 | 4.35 |
| 6 | The neatness of the health care service provider fascinates me. | 4.06 | 4.18 | 4.17 | 4.12 |
| 7 | The responsiveness of the health care service provider. | 3.85 | 3.82 | 4.00 | 4.29 |
| 8 | The mindfulness of the health care service provider. | 3.97 | 3.93 | 4.09 | 4.29 |
| 9 | The understanding of the health care service provider. | 4.07 | 4.00 | 4.09 | 4.41 |

Source: Field Survey, 2016

The result in Table 4.2.6c show the customers rating of how humanic clues influence switching restraint across the four age groups of the respondents. From the result as shown in Table 4.2.6c, it is evident that all the respondents have similar response pattern on the levels of agreement on statements relating to the influence of humanic clues on switching restraint. Notably, the result reveal positive responses concerning all the statements related to the influence of humanic clues on switching restraint as each of the nine statements has mean scores greater than 3.01 on a 5-Likert type scale. These indicate that all the age groups of the respondents are likely to be loyal to the organisation as a result of the actions, behaviour and expressions of their employees in the delivery of healthcare services. Findings from Table 4.2.6c also reveal that there are slight differences in the responses of the respondents in all age groups on the influence of humanic clues on switching restraint. However, it would appear that all age groups sampled are likely to respond similarly to the influence of humanic clues on switching restraint when it comes to healthcare services.

**TABLE 4.2.6d: The relationship between their experience and customer satisfaction**

| S/N | **Components of the Relationship between Customer Experience and Satisfaction** | **18-30**  **Years** | **31-40**  **Years** | **41-50**  **years** | **51 years+** |
| --- | --- | --- | --- | --- | --- |
|  |  | **Mean scores** | **Mean scores** | **Mean scores** | **Mean scores** |
| 1 | The reliability of the health care services makes me contented. | 4.18 | 4.09 | 4.07 | 4.35 |
| 2 | The right diagnosis of illness by the health care organisation | 4.01 | 4.06 | 4.16 | 4.35 |
| 3 | The right treatment of illness by the health care organisation enhances my relief. | 4.19 | 4.12 | 4.20 | 4.29 |
| 4 | The effective verbal communication of the health care service . | 3.99 | 4.03 | 4.09 | 4.59 |
| 5 | The non-verbal communication of the health care service enhances my gratification. | 3.54 | 3.64 | 3.79 | 4.12 |
| 6 | Respecting my wishes makes me satisfied. | 4.00 | 3.89 | 4.11 | 3.88 |
| 7 | Showing professionally appropriate behaviour by the health care service provider | 4.12 | 4.12 | 4.37 | 4.18 |
| 8 | Maintaining patient privacy enhances customer’s trust. | 4.09 | 4.15 | 4.29 | 4.29 |
| 9 | The aroma of the health care organisation makes me satisfied | 3.64 | 3.74 | 3.94 | 3.76 |
| 10 | The cleanliness of the health care organisation satisfies me | 4.17 | 4.03 | 4.13 | 4.29 |

Source: Field Survey, 2016

Ten items were used to investigate customers’ perception of the relationship between their experience and satisfaction with healthcare services offered by the four hospitals sampled based on the age of the respondents. From the result in Table 4.2.6d, it is obvious that the respondents in the four age brackets sampled have similar response pattern on the relationship between customer experience and satisfaction with healthcare services. The result also revealed positive responses concerning all the ten statements related to the relationship between customer experience and customer satisfaction as each of the statements has mean scores greater than the 3.01 on a 5-likert type scale.

**TABLE 4.2.6e: Buyers’ psychological factors moderate the relationship between CEM & customer loyalty in health care.**

| S/N | **Buyers’ Psychological Factors and the Relationship between CEM & Loyalty** | **18-30**  **years** | **31-40**  **Years** | **41-50**  **years** | **51 years+** |
| --- | --- | --- | --- | --- | --- |
|  |  | **Mean scores** | **Mean scores** | **Mean scores** | **Mean scores** |
| 1 | My perception about the reliability of services. | 4.13 | 4.14 | 4.30 | 4.18 |
| 2 | My interpretation of the right diagnosis of illness. | 4.10 | 3.98 | 4.16 | 4.29 |
| 3 | My motivation about the quality of health care services. | 4.16 | 3.97 | 4.20 | 4.29 |
| 4 | My experience with the behaviour of the health care service provider. | 4.04 | 3.98 | 4.13 | 4.35 |
| 5 | The belief I have concerning the right treatment of illness provided. | 4.07 | 3.95 | 4.13 | 4.24 |
| 6 | The maintenance of patient privacy by the healthcare organisation | 3.86 | 4.04 | 4.07 | 4.18 |
| 7 | The aroma/odor of the healthcare organisation. | 3.61 | 3.62 | 3.94 | 3.53 |
| 8 | My view about the effective verbal communication of the healthcare service provider. | 4.02 | 3.95 | 4.16 | 4.06 |
| 9 | My knowledge about the cleanliness of heath care organisation. | 3.95 | 4.11 | 4.16 | 4.00 |
| 10 | My interpretation of the tone of voice of the health care service provider. | 3.82 | 3.82 | 4.00 | 3.94 |
| 11 | My personality determines how I view the health care services provided | 4.15 | 4.11 | 4.16 | 4.12 |

Source: Field Survey, 2016

The result in Table 4.2.6e shows the customers’ view on how buyers’ psychological factors moderate the relationship between customer experience management and customer loyalty across the four age groups of the respondents. The result generally shows positive responses on all the statements related to how buyers’ psychological factors moderate the relationship between customer experience management and customer loyalty. Table 4.2.6e also reveals that the perception by those within the age bracket of (41-50) years have the highest mean scores of 4.30, this was followed by the respondents interpretation of the right diagnosis of illness and the motivation of customers about the quality of services with mean score of 4.29. This result is an indication that all age groupings of the respondents are in agreement on all the eleven statements concerning how buyers’ psychological factors moderate the relationship between customer experience management and customer loyalty.

## **4.2.7 CUSTOMERS’ PERCEPTION OF RESEARCH OBJECTIVES BASED ON EDUCATIONAL BACKGROUND OF THE RESPONDENTS**

**TABLE 4.2.7a: Customers’ Perception roles of functional clues on repeat purchase**

**actions according to educational qualification**

| **S/N** | **Components of Functional Clues on Repeat Purchase** | **WAEC** | **B.Sc** | **M.Sc/MBA** | **Others** |
| --- | --- | --- | --- | --- | --- |
|  |  | **Mean scores** | **Mean scores** | **Mean scores** | **Mean scores** |
| 1 | The reliability of the health care services. | 4.13 | 4.07 | 4.12 | 3.30 |
| 2 | The competence of the health care service. | 4.33 | 4.22 | 3.99 | 3.57 |
| 3 | The right diagnosis of the health care organisation. | 4.21 | 4.13 | 4.04 | 3.52 |
| 4 | Administration of the right treatment by the health care organisation. | 4.13 | 4.11 | 4.06 | 3.26 |
| 5 | The assured service of the health care organisation. | 4.21 | 4.08 | 4.01 | 3.30 |
| 6 | The procedures of the health care services. | 4.05 | 3.87 | 3.84 | 3.26 |
| 7 | The reliability of the continuous services. | 4.03 | 4.07 | 3.99 | 3.43 |
| 8 | The efficacy of the health care service. | 4.03 | 3.99 | 4.00 | 3.52 |
| 9 | The self-reliance of the health care service organisation. | 4.10 | 4.03 | 3.90 | 3.43 |
| 10 | The practicality of the healthcare services. | 3.90 | 3.92 | 3.93 | 3.35 |

Source: Field Survey, 2016

Table 4.2.7a shows the result of customers’ responses on the roles of functional clues on repeat purchase actions based on the educational background of the respondents. It is obvious from Table 4.2.7a that there are positive responses concerning all the statements related to the roles of functional clues on repeat purchase actions as each of the ten statements has mean scores greater than 3.01 on a 5-likert type scale. The findings indicate that the customers of all the hospitals sampled gave positive responses to all the statements. Going by the result, it can be inferred that functional clues play significant role in eliciting repeat purchase actions from customers of health institutions investigated.

**TABLE 4.2.7b: Customers’ perception of the influence of mechanic clues on brand**

**insistence of healthcare services**

| **S/N** | **Components of Mechanic Clues and Brand Insistence** | **WAEC** | **B.Sc** | **M.Sc/MBA** | **Others** |
| --- | --- | --- | --- | --- | --- |
|  |  | **Mean scores** | **Mean scores** | **Mean scores** | **Mean scores** |
| 1 | The physical appearance of the health care organisation | 3.85 | 4.13 | 3.87 | 4.13 |
| 2 | The conducive ambience of the health care organisation | 3.90 | 3.99 | 4.00 | 3.83 |
| 3 | The furniture and fitting of the health care organisation | 3.46 | 3.70 | 3.70 | 3.30 |
| 4 | The facilities of the health care organisation. | 4.10 | 4.06 | 4.06 | 3.83 |
| 5 | The lighting of the health care organisation. | 3.95 | 3.94 | 4.28 | 3.65 |
| 6 | The cleanliness of the health care organisation. | 4.15 | 4.31 | 4.21 | 3.91 |
| 7 | The comfort of the health care lobby/reception area. | 3.85 | 4.04 | 4.07 | 3.83 |
| 8 | The convenient location of the health care organisation. | 4.13 | 3.96 | 3.99 | 3.57 |
| 9 | The aroma of the health care organisation | 3.15 | 3.49 | 3.57 | 3.43 |

Source: Field Survey, 2016

Nine items constructed in statement formats were used to investigate the respondents’ opinion on the influence of mechanic clues on brand insistence across the four groups of customers with different educational qualifications. The result as presented in Table 4.2.7b shows that the respondents of different educational qualifications have similar response pattern on the effect of mechanic clues on brand insistence. The result also reveals positive responses on all the statements related to the influence of mechanic clues on brand insistence as each of the nine statements has mean score greater than 3.01 on a 5-likert type scale. The findings here indicate that exceptional consideration should be given to the cleanliness of the healthcare organisation as it has the highest mean scores for the respondents with O’ Level, Bachelor and Masters degrees, for those with other qualifications, they were of the view that the physical appearance of the health facilities has more influence on brand insistence.

**TABLE 4.2.7c: Customers’ perception of the influence of humanic clues on switching**

**restraint**

| S/N | **Components of Humanic Clues and Switching Restraint** | **WAEC** | **B.Sc** | **M.Sc/MBA** | **Others** |
| --- | --- | --- | --- | --- | --- |
|  |  | **Mean scores** | **Mean scores** | **Mean scores** | **Mean scores** |
| 1 | The friendly actions of the health care service providers. | 4.33 | 4.14 | 4.10 | 3.96 |
| 2 | The caring expression of the health care service providers. | 4.13 | 4.11 | 3.99 | 3.78 |
| 3 | The tone of voice of the health care service provider pleases me. | 4.03 | 4.03 | 3.93 | 3.52 |
| 4 | The body language of the health care service provider. | 3.92 | 3.71 | 3.87 | 3.43 |
| 5 | The respect and courtesy from the health care service provider. | 4.28 | 4.09 | 4.04 | 3.65 |
| 6 | The neatness of the health care service provider fascinates me. | 4.15 | 4.17 | 4.07 | 4.04 |
| 7 | The responsiveness of the health care service provider. | 4.10 | 3.90 | 3.91 | 3.35 |
| 8 | The mindfulness of the health care service provider. | 4.03 | 4.03 | 3.96 | 3.61 |
| 9 | The understanding of the health care service provider. | 4.18 | 4.06 | 4.09 | 3.65 |

Source: Field Survey, 2016

Table 4.2.7c is the presentation of the result of the analysis of data how humanic clues influence switching restraint as perceived by the respondents of different educational background of the respondents. From the result presented in Table 4.2.7c, it is evident that all the respondents have similar response pattern on the influence of humanic clues on switching restraint and that positive responses were obtained in the statements related to this. As can be seen in the result, all the mean scores for each statement are more than the 3.01 on a 5-likert type scale. The findings (Table 4.2.7c) reveal that there are slight differences in the responses of the respondents across the different educational qualification as they relate to the influence of humanic clues on switching restraint. For example the respondents with the highest educational qualifications of O’ Level WAEC, B.Sc and M.Sc/MBA were of the view that the friendly actions of the healthcare service providers with the highest mean scores of 4.33, 4.14 and 4.10 respectively, has the most influence on switching. On the other hand, the respondents with other qualifications indicated that the neatness of the healthcare service providers with mean score of 4.04 has the most influence on switching restraints.

**TABLE 4.2.7d: Customers’ perception of the relationship between customer**

**experience and customer satisfaction in health care services**

| S/N | **Components of Customer Experience and Satisfaction** | **WAEC** | **B.Sc** | **M.Sc/MBA** | **Others** |
| --- | --- | --- | --- | --- | --- |
|  |  | **Mean scores** | **Mean scores** | **Mean scores** | **Mean scores** |
| 1 | The reliability of the health care services makes me contented. | 4.31 | 4.16 | 4.06 | 3.61 |
| 2 | The right diagnosis of illness by the health care organisation | 4.15 | 4.13 | 4.01 | 3.65 |
| 3 | The right treatment of illness by the health care organisation enhances my relief. | 4.28 | 4.23 | 4.04 | 3.65 |
| 4 | The effective verbal communication of the health care service . | 4.33 | 4.05 | 4.03 | 3.74 |
| 5 | The non-verbal communication of the health care service enhances my gratification. | 3.82 | 3.67 | 3.70 | 3.13 |
| 6 | Respecting my wishes makes me satisfied. | 4.05 | 4.02 | 3.94 | 3.35 |
| 7 | Showing professionally appropriate behaviour by the health care service provider | 4.13 | 4.17 | 4.30 | 3.91 |
| 8 | Maintaining patient privacy enhances customer’s trust. | 4.23 | 4.18 | 4.19 | 3.87 |
| 9 | The aroma of the health care organisation makes me satisfied | 3.62 | 3.75 | 3.90 | 3.57 |
| 10 | The cleanliness of the health care organisation satisfies me | 4.21 | 4.09 | 4.16 | 3.83 |

Source: Field Survey, 2016

The study also investigated the customers’ views on the relationship between their experience and satisfaction with healthcare services based on their educational qualifications. The result in Table 4.2.7d reveals that the respondents with different educational backgrounds have similar response pattern on the relationship between their experience and satisfaction with the healthcare services. The result shows positive responses concerning all the statements on this as all the mean scores are more than the 3.01 on a 5-likert type scale. However, Table 4.2.7d indicates that those with O’Level (WAEC) as their highest educational qualification were of the view that effective verbal communication of the healthcare service providers with the mean score of 4.33 was the most visible evidence of the relationship between their experience and satisfaction with healthcare service. Those with bachelor degree indicated that it was the right treatment of illness (4.23) and those with master’s degrees and other qualification said it was showing professionally appropriate behaviour by the healthcare service providers.

**TABLE 4.2.7e: Customers’ perception of how psychological factors moderate the**

**relationship between CEM & loyalty.**

| S/N | **Components of Psychological Factors, CEM and Loyalty** | **WAEC** | **B.Sc** | **M.Sc/MBA** | **Others** |
| --- | --- | --- | --- | --- | --- |
|  |  | **Mean scores** | **Mean scores** | **Mean scores** | **Mean scores** |
| 1 | My perception about the reliability of services. | 4.10 | 4.23 | 4.18 | 3.61 |
| 2 | My interpretation of the right diagnosis of illness. | 3.97 | 4.11 | 4.09 | 3.61 |
| 3 | My motivation about the quality of health care services. | 4.26 | 4.09 | 4.09 | 3.70 |
| 4 | My experience with the behaviour of the health care service provider. | 4.18 | 4.04 | 4.12 | 3.57 |
| 5 | The belief I have concerning the right treatment of illness provided. | 4.00 | 4.03 | 4.16 | 3.78 |
| 6 | The maintenance of patient privacy by the healthcare organisation | 4.00 | 3.98 | 4.15 | 3.83 |
| 7 | The aroma/odor of the healthcare organisation. | 3.56 | 3.65 | 3.90 | 3.52 |
| 8 | My view about the effective verbal communication of the healthcare service provider. | 4.21 | 4.01 | 4.00 | 3.74 |
| 9 | My knowledge about the cleanliness of heath care organisation. | 4.05 | 4.09 | 4.10 | 3.78 |
| 10 | My interpretation of the tone of voice of the health care service provider. | 3.97 | 3.85 | 3.90 | 3.65 |
| 11 | My personality determines how I view the health care services provided | 4.23 | 4.12 | 4.20 | 3.83 |

Source: Field Survey, 2016

Table 4.2.7e shows the result of the analysis of how buyers’ psychological factors moderate the relationship between customer experience management and customer loyalty across the different levels of qualifications of the respondents. Generally, the result reveals positive responses on all the statements related to this. However, Table 4.2.7e reveals that the consumers’ motivation about the quality of services for those respondents with WAEC has the highest mean score of 4.26, followed by the consumer perception about the reliability of healthcare services for those respondents with B.Sc degree and M.Sc/MBA with mean scores of 4.23 and 4.18 respectively. In all, this result is an indication that the respondents of different educational background agree with all the statements concerning how buyers’ psychological factors moderate the relationship between customer experience management and customer loyalty in the healthcare organisations investigated in this research.

## **4.2.8 CONSUMERS’ PERCEPTION OF RESEARCH OBJECTIVES BASED ON MARITAL STATUS OF THE CUSTOMERS**

**TABLE 4.2.8a: Consumers’ perception of the roles of functional clues on repeat**

**purchase of healthcare services**

| S/N | **Components of Functional Clues and repeat Purchase** | **Single** | **Married** | **Divorced** |
| --- | --- | --- | --- | --- |
|  |  | **Mean scores** | **Mean scores** | **Mean scores** |
| 1 | The reliability of the health care services. | 4.01 | 4.04 | 4.50 |
| 2 | The competence of the health care service. | 4.25 | 4.10 | 4.33 |
| 3 | The right diagnosis of the health care organisation. | 4.11 | 4.07 | 4.00 |
| 4 | Administration of the right treatment by the health care organisation. | 3.98 | 4.08 | 4.00 |
| 5 | The assured service of the health care organisation. | 4.03 | 4.02 | 4.67 |
| 6 | The procedures of the health care services. | 3.76 | 3.88 | 4.00 |
| 7 | The reliability of the continuous services. | 4.02 | 4.00 | 4.17 |
| 8 | The efficacy of the health care service. | 4.00 | 3.94 | 4.17 |
| 9 | The self-reliance of the health care service organisation. | 3.93 | 3.99 | 4.17 |
| 10 | The practicality of the healthcare services. | 3.95 | 3.85 | 4.17 |

Source: Field Survey, 2016

Ten items in statements format were used to investigate the customers’ perception of the roles of functional clues on repeat purchase of healthcare services. Table 4.2.8a, shows the analysis of customers’ responses on the role of functional clues on repeat purchase actions based on their marital status. The result reveals positive responses on all the statements related to this as all the mean scores for the ten items are more than the 3.01 on a 5-likert type scale. The findings on Table 4.2.8a show that the customers of all the hospitals who are in marriage and those not in marriage relationships gave positive responses to the statements. Going by this result, it is evident that functional clues play significant role in eliciting repeat purchase actions from customers of the four hospitals irrespective of their marital status.

**TABLE 4.2.8b: Customers’ perception of the influence of mechanic clues on brand insistence in healthcare services**

| S/N | **Components of Mechanic Clues and Brand Insistence** | **Single** | **Married** | **Divorced** |
| --- | --- | --- | --- | --- |
|  |  | **Mean scores** | **Mean scores** | **Mean scores** |
| 1 | The physical appearance of the health care organisation | 4.04 | 4.05 | 4.50 |
| 2 | The conducive ambience of the health care organisation | 4.03 | 3.94 | 4.17 |
| 3 | The furniture and fitting of the health care organisation | 3.48 | 3.71 | 4.00 |
| 4 | The facilities of the health care organisation. | 4.10 | 4.02 | 4.17 |
| 5 | The lighting of the health care organisation. | 4.10 | 3.93 | 4.00 |
| 6 | The cleanliness of the health care organisation. | 4.22 | 4.27 | 4.00 |
| 7 | The comfort of the health care lobby/reception area. | 3.95 | 4.03 | 4.33 |
| 8 | The convenient location of the health care organisation. | 3.91 | 3.97 | 4.17 |
| 9 | The aroma of the health care organisation | 3.23 | 3.55 | 4.33 |

Source: Field Survey, 2016

Table 4.2.8b shows the result on the respondents’ opinion on the influence of mechanic clues on brand insistence based on the marital status of the respondents. The result (Table 4.2.8b) clearly show that the respondents of different marital status have similar views on the influence of mechanic clues on brand insistence. Although positive responses were obtained on all the statements investigated as each of the nine items emerged with the mean scores greater than the 3.01 on a 5-likert type scale, the respondents who had never married before and those in marriage relationships at the time of the survey opined that the cleanliness of healthcare facilities with mean scores of 4.22 and 4.27, respectively had the most influence on brand insistence in healthcare services. On the other hand, the respondents who were divorced viewed the physical appearance of the healthcare facilities as having the most influence on brand insistence in healthcare services.

**TABLE 4.2.8c: Consumers’ perception of the influence of humanic clues on switching restraint**

| S/n | **Components of Humanic Clues and Switching Restraint** | **Single** | **Married** | **Divorced** |
| --- | --- | --- | --- | --- |
|  |  | **Mean scores** | **Mean scores** | **Mean scores** |
| 1 | The friendly actions of the health care service providers make me loyal to the organisation | 4.13 | 4.14 | 4.83 |
| 2 | The caring expression of the health care service providers. | 4.05 | 4.08 | 4.33 |
| 3 | The tone of voice of the health care service provider pleases me | 3.75 | 4.10 | 3.50 |
| 4 | The body language of the health care service provider encourages me to patronize. | 3.75 | 3.75 | 3.67 |
| 5 | The respect and courtesy from the health care service provider reinforces my patronage. | 4.07 | 4.09 | 3.67 |
| 6 | The neatness of the health care service provider fascinates me. | 4.09 | 4.17 | 3.83 |
| 7 | The responsiveness of the health care service provider makes me restrain switching from the organisation. | 3.94 | 3.87 | 3.67 |
| 8 | The mindfulness of the health care service provider makes me satisfied | 3.91 | 4.03 | 3.50 |
| 9 | The understanding of the health care service provider enhances my commitment to the organisation. | 4.03 | 4.08 | 3.50 |

Source: Researcher’s Field Survey, 2016

The result in Table 4.2.8c highlight the customers’ perception of how humanic clues influence switching restraint among married and unmarried respondents. From the result presented in Table 4.2.8c, it is evident that all the respondents irrespective of their marital status have similar views on the influence of humanic clues on switching restraint. This based on the fact that positive responses were obtained on all the statements related to the influence of humanic clues on switching restraint and the items mean scores have more than the 3.01 on a 5-likert type scale. Further, the result in Table 4.2.8c revealed that there are slight differences in the responses of the respondents on the influence of humanic clues on switching restraint across the three groups of customers who took part in the survey. Specifically, whereas, those who were in marriage relationship viewed the neatness of the healthcare service providers (4.17) as having the most influence on switching restraint, those who were not in marriage relationship considered the friendly actions of the healthcare service providers with mean scores of 4.13 for the singles and 4.83 for the divorced as the aspect of humanic clues with the most influence on switching restraints.

**TABLE 4.2.8d: Relationship between customer experience and customer satisfaction**

**in the health care sector**

| S/N | **Components of Customer Experience and Customer Satisfaction** | **Single** | **Married** | **Divorced** |
| --- | --- | --- | --- | --- |
|  |  | **Mean scores** | **Mean scores** | **Mean scores** |
| 1 | The reliability of the health care services makes me contented. | 4.18 | 4.08 | 4.67 |
| 2 | The right diagnosis of illness by the health care organisation makes me completely happy with the organisation. | 4.04 | 4.09 | 4.33 |
| 3 | The right treatment of illness by the health care organisation enhances my relief. | 4.22 | 4.14 | 4.33 |
| 4 | The effective verbal communication of the health care service. | 3.97 | 4.09 | 4.17 |
| 5 | The non-verbal communication of the health care service enhances my gratification. | 3.65 | 3.65 | 4.33 |
| 6 | Respecting my wishes makes me satisfied. | 4.01 | 3.92 | 4.83 |
| 7 | Showing professionally appropriate behaviour by the health care service provider | 4.23 | 4.13 | 4.67 |
| 8 | Maintaining patient privacy enhances customer’s trust. | 4.15 | 4.16 | 5.00 |
| 9 | The aroma of the health care organisation makes me satisfied | 3.74 | 3.75 | 4.33 |
| 10 | The cleanliness of the health care organisation satisfies me | 4.20 | 4.04 | 4.67 |

Source: Field Survey, 2016

The views of the respondents in marriage and those not in marriage relationships on the relationship between customer experience and customer satisfaction are presented in Table 4.2.8d. Going by the result (Table 4.2.8d). It is obvious that the respondent have similar pattern of responses on the relationship between customer experience and satisfaction with healthcare services. The result revealed positive responses on all the statements related to the relationship between customer experience and satisfaction with healthcare services as mean scores of the ten items are more than the 3.01 on a 5-likert type scale. However, there are differences among the three categories of respondents on which aspects of captures most the relationship between customer experience and satisfaction with healthcare services in the four private hospitals investigated in this study.

| S/n | **Components of Buyers’ Psychological Factors and the Relation-ship between CEM and Loyalty** | **Single** | **Married** | **Divorced** |
| --- | --- | --- | --- | --- |
|  |  | **Mean scores** | **Mean scores** | **Mean scores** |
| 1 | My perception about the reliability of services provided | 4.21 | 4.14 | 4.50 |
| 2 | My interpretation of the right diagnosis of illness. | 4.06 | 4.06 | 4.33 |
| 3 | My motivation about the quality of health care services. | 4.18 | 4.03 | 4.33 |
| 4 | My experience with the behaviour of the health care service provider. | 4.12 | 3.99 | 4.67 |
| 5 | The belief I have concerning the right treatment of illness provided by the organisation. | 4.11 | 3.99 | 4.50 |
| 6 | The maintenance of patient privacy by the healthcare organisation. | 3.95 | 4.02 | 4.50 |
| 7 | The aroma/odor of the healthcare organisation. | 3.55 | 3.73 | 3.83 |
| 8 | My view about the effective verbal communication of the healthcare service provider. | 4.01 | 4.02 | 3.83 |
| 9 | My knowledge about the cleanliness of heath care organisation surroundings. | 4.03 | 4.08 | 4.33 |
| 10 | My interpretation of the tone of voice of the health care service provider. | 3.85 | 3.86 | 4.17 |
| 11 | My personality determines how I view the health care services provided. | 4.18 | 4.10 | 4.17 |

**TABLE 4.2.8e: Customers; perception of how their psychological factors moderate the**

**relationship between CEM and loyalty**

Source: Researcher’s Field Survey, 2016

Table 4.2.8e shows the result of the level of agreement by the customers of how the buyers’ psychological factors moderate the relationship between customer experience management and loyalty based on their marital status. This result reveals that experience with the behaviour of the health care service providers for those respondents in the divorced category has the highest mean scores of 4.67, followed by the perception of customers about the reliability of the services provided, belief about right treatment, and maintenance of patient privacy with mean scores of 4.50 for the same category of respondents. Those that were married and the singles’ were of the opinion that the perception of customers about the reliability of the services provided with mean scores of 4.14 and 4.21, respectively moderated most this relationship. This result is an indication that in spite of the marital status of the respondents, they all agreed with the statements concerning how buyers’ psychological factors moderate the relationship between customer experience management and loyalty in the healthcare sector of the study area.

**4.2.9 CUSTOMERS’ PERCEPTION OF RESEARCH OBJECTIVES BASED ON THE EMPLOYMENT STATUS OF RESPONDENTS**

**TABLE 4.2.9a: Customers’ perception of the roles of functional clues on repeat purchase across employment status of the respondents**

| **S/N** | **Components of Functional Clues and Repeat Purchase** | **Student** | **Employer** | **Employee** |
| --- | --- | --- | --- | --- |
|  |  | **Mean scores** | **Mean scores** | **Mean scores** |
| 1 | The reliability of the health care services. | 3.96 | 4.03 | 4.07 |
| 2 | The competence of the health care service. | 4.15 | 4.06 | 4.17 |
| 3 | The right diagnosis of the health care organisation. | 4.08 | 4.11 | 4.07 |
| 4 | Administration of the right treatment by the health care organisation. | 4.09 | 4.07 | 4.02 |
| 5 | The assured service of the health care organisation. | 3.99 | 3.97 | 4.08 |
| 6 | The procedures of the health care services. | 3.81 | 3.88 | 3.85 |
| 7 | The reliability of the continuous services. | 3.87 | 4.01 | 4.07 |
| 8 | The efficacy of the health care service. | 3.97 | 4.07 | 3.93 |
| 9 | The self-reliance of the health care service organisation. | 3.96 | 3.94 | 3.99 |
| 10 | The practicality of the healthcare services. | 4.01 | 3.99 | 3.79 |

Source: Field Survey, 2016

The analysis of customers’ responses on the role of functional clues on repeat purchase actions based on the employment status of the respondents is presented in Table 4.2.9a. The result (Table 4.2.9a.) reveals positive responses on all the statements related to this and all the statements show mean scores greater than the 3.01 on a 5-likert type scale. The findings on this show that the customers of all the hospitals irrespective of their employment status gave positive responses to the statements on the roles functional clues in eliciting repeat purchase actions from customers. However, there are differences on which aspect of functional clues have the most influence on repeat purchase actions among the students, employers and employees. Whereas the student and the employees viewed the competence of the healthcare service providers as playing the most significant role, the employers rated the right diagnosis as playing the most important role in eliciting repeat purchase actions from healthcare customers.

**TABLE 4.2.9b: Customers’ perception of the influence of mechanic clues on brand insistence of healthcare service**

| S/N | **Components of Mechanic Clues and Brand Insistence** | **Student** | **Employer** | **Employee** |
| --- | --- | --- | --- | --- |
|  |  | **Mean scores** | **Mean scores** | **Mean scores** |
| 1 | The physical appearance of the health care organisation | 4.05 | 4.07 | 4.04 |
| 2 | The conducive ambience of the health care organisation | 4.01 | 4.03 | 3.93 |
| 3 | The furniture and fitting of the health care organisation | 3.57 | 3.73 | 3.66 |
| 4 | The facilities of the health care organisation. | 4.16 | 4.12 | 3.98 |
| 5 | The lighting of the health care organisation. | 4.03 | 3.81 | 4.02 |
| 6 | The cleanliness of the health care organisation. | 4.19 | 4.33 | 4.25 |
| 7 | The comfort of the health care lobby/reception area. | 3.94 | 4.16 | 4.00 |
| 8 | The convenient location of the health care organisation. | 3.89 | 4.07 | 3.95 |
| 9 | The aroma of the health care organisation | 3.34 | 3.55 | 3.50 |

Source: Field Survey, 2016

The perceptions of the customers on the influence of mechanic clues on brand insistence was also investigated across the three categories of respondents based on their employment status. Based on the result presented in Table 4.2.9b, it is evident that irrespective of the occupation of respondents, they share views on the influence of mechanic clues on brand insistence. The result shows positive responses on all the nine statements related to the influence of mechanic clues on brand insistence and all the items mean scores have more than the 3.01 on a 5-likert type scale. Interestingly, among the students, employers and employees’ respondents, cleanliness of the healthcare facilities with mean scores of 4.19, 4.33 and 4.25, respectively, emerged as the component of mechanic clues that has the most influence on brand insistence in healthcare services in the four hospitals sampled in this study.

**TABLE 4.2.9c: Customers’ perception of the influence of humanic clues on switching restraint**

| S/N | **Components of Humanic Clues and Switching Restraint** | **Student** | **Employer** | **Employee** |
| --- | --- | --- | --- | --- |
|  |  | **Mean scores** | **Mean scores** | **Mean scores** |
| 1 | The friendly actions of the health care service providers. | 4.20 | 4.12 | 4.13 |
| 2 | The caring expression of the health care service providers. | 4.09 | 4.22 | 4.01 |
| 3 | The tone of voice of the health care service provider pleases me. | 4.16 | 3.84 | 3.95 |
| 4 | The body language of the health care service provider. | 3.77 | 3.61 | 3.78 |
| 5 | The respect and courtesy from the health care service provider. | 4.08 | 4.10 | 4.06 |
| 6 | The neatness of the health care service provider fascinates me. | 4.08 | 4.22 | 4.14 |
| 7 | The responsiveness of the health care service provider. | 4.07 | 3.91 | 3.79 |
| 8 | The mindfulness of the health care service provider. | 4.13 | 4.03 | 3.91 |
| 9 | The understanding of the health care service provider. | 4.27 | 4.09 | 3.94 |

Source: Field Survey, 2016

The result in Table 4.2.9c reveals the influence of humanic clues on switching restraints. The result shows positive responses on all the nine statements related to this. In fact, all the nine items have greater mean scores than the 3.01 on a 5-likert type scale. Findings in Table 4.2.9c concerning the students’ view reveal that healthcare service providers should pay special attention to understanding their patient as it has a mean score of 4.27. For the employers, they need to give special attention to the caring expression and the neatness of the healthcare service providers, both of which has mean scores of 4.22; and for the employees, emphasis should be given to friendly attitudes by the service providers to prevent this category of customers from switching to other healthcare service providers.

**TABLE4.2.9d: Customers’ perception of the relationship between customer**

**experience and customer satisfaction**

| S/N | **Components of Customer Experience and Satisfaction** | **Student** | **Employer** | **Employee** |
| --- | --- | --- | --- | --- |
|  |  | **Mean scores** | **Mean scores** | **Mean scores** |
| 1 | The reliability of the health care services makes me contented. | 4.21 | 4.21 | 4.05 |
| 2 | The right diagnosis of illness by the health care organisation makes me completely happy with the organisation. | 4.14 | 4.22 | 4.00 |
| 3 | The right treatment of illness by the health care organisation. | 4.32 | 4.27 | 4.06 |
| 4 | The effective verbal communication of the health care service enhances my gratification. | 4.00 | 4.22 | 4.03 |
| 5 | The non-verbal communication of the health care service enhances my gratification. | 3.64 | 3.60 | 3.69 |
| 6 | Respecting my wishes makes me satisfied. | 4.08 | 4.00 | 3.90 |
| 7 | Showing professionally appropriate behaviour by the health care service provider delights me | 4.14 | 4.19 | 4.18 |
| 8 | Maintaining patient privacy enhances customer’s trust. | 4.20 | 4.36 | 4.09 |
| 9 | The aroma of the health care organisation makes me satisfied | 3.68 | 3.87 | 3.75 |
| 10 | The cleanliness of the health care organisation satisfies me | 4.19 | 4.22 | 4.01 |

Source: Field Survey, 2016

The study also investigated the customers’ views on the relationship between their experience and satisfaction with healthcare services based on their employment status. The result in Table 4.2.9d reveals that the respondents with different educational backgrounds have similar response pattern on the relationship between their experience and satisfaction with the healthcare services. The result shows positive responses concerning all the statements on this as all the mean scores are more than the 3.01 on a 5-likert type scale. However, Findings in Table 4.2.9d show that there are differences among the three categories of respondents on which aspects of captures most the relationship between customer experience and satisfaction with healthcare services in the four private hospitals investigated in this study.

**TABLE 4.2.9e: Customers’ perception of how their psychological factors moderate the**

**relationship between CEM & loyalty.**

| S/N | **Components of Buyers’ Psychological Factor on the Relationship between CEM and Loyalty** | **Student** | **Employer** | **Employee** |
| --- | --- | --- | --- | --- |
|  |  | **Mean scores** | **Mean scores** | **Mean scores** |
| 1 | My perception about the reliability of services provided | 4.20 | 4.24 | 4.13 |
| 2 | My interpretation of the right diagnosis of illness. | 4.08 | 4.19 | 4.01 |
| 3 | My motivation about the quality of health care services. | 4.25 | 4.06 | 4.01 |
| 4 | My experience with the behaviour of the health care service provider. | 4.12 | 4.09 | 3.99 |
| 5 | The belief I have concerning the right treatment of illness provided by the organisation. | 4.14 | 3.99 | 4.00 |
| 6 | The maintenance of patient privacy by the healthcare organisation. | 3.89 | 4.09 | 4.02 |
| 7 | The aroma/odor of the healthcare organisation. | 3.53 | 3.72 | 3.73 |
| 8 | My view about the effective verbal communication of the healthcare service provider. | 3.98 | 4.03 | 4.02 |
| 9 | My knowledge about the cleanliness of heath care organisation surroundings. | 4.03 | 4.16 | 4.05 |
| 10 | My interpretation of the tone of voice of the health care service provider. | 3.88 | 3.81 | 3.87 |
| 11 | My personality determines how I view the health care services provided. | 4.24 | 4.09 | 4.09 |

Source: Researcher’s Field Survey, 2016

Table 4.2.9e shows the result of the level of agreement by the customers of how the buyers’ psychological factors moderate the relationship between customer experience management and loyalty based on their employment status. This result (Table 4.2.9e) reveals that the employer and the employee category of respondents were of the opinion that the perception of customers about the reliability of the services provided with mean scores of 4.24 and 4.13, respectively moderated most this relationship. Furthermore, this result reveals that their motivation about the quality of health care services for those respondents in the students’ category has the highest mean scores of 4.25. This result is an indication that in spite of the employment status of the respondents, they all agreed with the statements concerning how buyers’ psychological factors moderate the relationship between customer experience management and loyalty in the healthcare sector of the study area.

## **4.2.10 CONSUMERS’ PERCEPTION OF RESEARCH OBJECTIVES BASED ON CLASS OF CUSTOMERS**

**TABLE 4.2.10a: Customers’ perception of the roles of functional clues on repeat purchase**

| **Components of Functional Clues and Repeat Purchase** | **Corporate Customer** | **Private Individual** |
| --- | --- | --- |
|  | **Mean scores** | **Mean scores** |
| The reliability of the health care services. | 3.94 | 4.17 |
| The competence of the health care service. | 4.04 | 4.30 |
| The right diagnosis of the health care organisation. | 4.04 | 4.15 |
| Administration of the right treatment by the health care organisation. | 4.03 | 4.07 |
| The assured service of the health care organisation. | 3.99 | 4.11 |
| The procedures of the health care services. | 3.84 | 3.85 |
| The reliability of the continuous services. | 3.94 | 4.12 |
| The efficacy of the health care service. | 3.91 | 4.05 |
| The self-reliance of the health care service organisation. | 3.92 | 4.05 |
| The practicality of the healthcare services. | 3.80 | 4.00 |

Source: Researcher’s Field Survey, 2016

| Table 4.2.10a shows that both the corporate customer and private individual have a similar response pattern to the roles of functional clues on repeat purchase. The Table also reveals positive responses concerning all the statements related to the roles of functional clues on repeat purchase as all the mean scores are more than the 3.01 on a 5-likert type scale. These findings pointed out that the class of customers with the organisation makes no significant difference as they are likely to make a repeat purchase as a result of the competence of the healthcare service delivery. Findings from the Table 4.2.10a reveal that class of customers with the organisation have the same view on the roles functional clues plays in eliciting repeat purchase behaviour of the customers. As both corporate customer and private individual are likely to react similarly to functional clues as delivered by the organisation. |
| --- |

**TABLE 4.2.10b: Customers’ perception of the influence of mechanic clues on brand insistence**

| **Components of Mechanic Clues and Brand Insistence** | **Corporate Customer** | **Private Individual** |
| --- | --- | --- |
|  | **Mean scores** | **Mean scores** |
| The physical appearance of the health care organisation | 4.05 | 4.06 |
| The conducive ambience of the health care organisation | 3.94 | 4.01 |
| The furniture and fitting of the health care organisation | 3.69 | 3.59 |
| The facilities of the health care organisation. | 3.98 | 4.15 |
| The lighting of the health care organisation. | 4.07 | 3.85 |
| The cleanliness of the health care organisation. | 4.19 | 4.32 |
| The comfort of the health care lobby/reception area. | 4.00 | 4.03 |
| The convenient location of the health care organisation. | 3.96 | 3.95 |
| The aroma of the health care organisation | 3.47 | 3.46 |

| Source: Researcher’s Field Survey, 2016    The Table 4.2.10b was designed to measure respondents’ opinion on the effect on mechanic clues on brand insistence based on class of customers. From the result revealed and shown in Table 4.2.10b, it is obvious that both corporate customers and private individuals have a similar response pattern on the influence of mechanic clues on brand insistence. The Table also reveals positive responses concerning all the statements related to the influence of mechanic clues on brand insistence as all the mean scores are more than the 3.01 on a 5-likert type scale. These findings in (Table 4.2.10b) indicate that both classes of customers are likely to insist on brand as a result of the cleanliness of the healthcare organisation as both corporate and private customers viewed it as playing the most significant influence on brand insistence with the highest mean scores of 4.19 and 4.32 respectively. |
| --- |

**TABLE 4.2.10c: Customers’ perception of the influence of humanic clues on switching restraint**

| **Components of Humanic Clues and Switching Restraint** | **Corporate Customer** | **Private Individual** |
| --- | --- | --- |
|  | **Mean scores** | **Mean scores** |
| The friendly actions of the health care service providers. | 4.09 | 4.23 |
| The caring expression of the health care service providers. | 4.02 | 4.14 |
| The tone of voice of the health care service provider pleases me. | 4.11 | 3.79 |
| The body language of the health care service provider. | 3.73 | 3.77 |
| The respect and courtesy from the health care service provider. | 4.08 | 4.07 |
| The neatness of the health care service provider fascinates me. | 4.12 | 4.18 |
| The responsiveness of the health care service provider. | 3.89 | 3.88 |
| The mindfulness of the health care service provider. | 3.90 | 4.11 |
| The understanding of the health care service provider. | 4.05 | 4.07 |

| Source: Researcher’s Field Survey, 2016  Result in Table 4.2.10c underscore how humanic clues influence switching restraint based on respondents’ status with the firm. The result reveals positive responses on all the statements related to the influence of humanic clues on switching restraint as all the mean scores are greater than 3.01 on a 5-likert type scale. These findings show that the corporate customer are of the opinion that the neatness of the health care service providers plays most significant role with a mean score of 4.12, while the private customers viewed the friendly actions of the healthcare service providers as playing the most significant role in influencing their switching restraint to the organisation with the highest mean score of 4.23. |
| --- |

**TABLE 4.2.10d: Relationship between customer experience and customer satisfaction**

**in the health care sector**

| **Components of Customer Experience and Satisfaction** | **Corporate Customer** | **Private Individual** |
| --- | --- | --- |
|  | **Mean scores** | **Mean scores** |
| The reliability of the health care services makes me contented. | 4.04 | 4.25 |
| The right diagnosis of illness by the health care organisation makes me completely happy | 4.00 | 4.20 |
| The right treatment of illness by the health care organisation enhances my relief. | 4.05 | 4.34 |
| The effective verbal communication of the health care service enhances my gratification. | 3.97 | 4.19 |
| The non-verbal communication of the health care service enhances my gratification. | 3.63 | 3.70 |
| Respecting my wishes makes me satisfied. | 3.85 | 4.13 |
| Showing professionally appropriate behaviour by the health care service provider delights me | 4.14 | 4.21 |
| Maintaining patient privacy enhances customer’s trust. | 4.13 | 4.22 |
| The aroma of the health care organisation makes me satisfied | 3.75 | 3.76 |
| The cleanliness of the health care organisation satisfies me | 4.00 | 4.24 |

| Source: Researcher’s Field Survey, 2016  The result in Table 4.2.10d highlight the customers’ perception of the relationship between customer experience and satisfaction based on category of customers with the firm. The result in Table 4.2.10d reveals positive responses concerning all the statements related to the relationship between customer experience and satisfaction as all the mean scores are more than the 3.01 on a 5-likert type scale. However, These findings signify that the corporate customers are of the view that showing professionally appropriate behaviour by the health care service providers (4.14) has the most influence on satisfaction whereas, the private customers considered the reliability of the healthcare services with mean scores of 4.25 as an aspect of customer experience with the most influence on satisfaction. This is an indication that both categories of customers are likely to be satisfied with the service of the organisation as a result of customer experience strategies employ by the organisation. |
| --- |

**TABLE 4.2.10e: How buyers’ psychological factors moderate the relationship between**

**CEM & customer loyalty.**

| **Components of Buyers’ Psychological Factors on the Relationship between CEM and Loyalty** | **Corporate Customer** | **Private Individual** |
| --- | --- | --- |
|  | **Mean scores** | **Mean scores** |
| My perception about the reliability of services provided | 4.11 | 4.26 |
| My interpretation of the right diagnosis of illness. | 3.99 | 4.17 |
| My motivation about the quality of health care services. | 3.97 | 4.25 |
| My experience with the behaviour of the health care service provider. | 4.03 | 4.06 |
| The belief I have concerning the right treatment of illness provided by the organisation. | 3.98 | 4.11 |
| The maintenance of patient privacy by the healthcare organisation. | 3.94 | 4.10 |
| The aroma/odor of the healthcare organisation. | 3.64 | 3.72 |
| My view about the effective verbal communication of the healthcare service provider. | 3.94 | 4.12 |
| My knowledge about the cleanliness of heath care organisation surroundings. | 4.02 | 4.13 |
| My interpretation of the tone of voice of the health care service provider. | 3.84 | 3.89 |
| My personality determines how I view the health care services provided. | 4.10 | 4.18 |

Source: Researcher’s Field Survey, 2016

The Table 4.2.10e was designed to find out how the buyers’ psychological factors moderate the relationship between customer experience management and customer loyalty based on respondents’ status with the firms. From the result revealed and presented, it is obvious that both corporate customer and private individual have similar response pattern on how the buyers’ psychological factors moderate the relationship between customer experience management and customer loyalty as the Table also revealed positive responses concerning all the statements related to how buyers’ psychological factors moderate the relationship between customer experience management and customer loyalty. Based on the result presented in Table 4.2.10e, it is evident that both customers considered their perception about the reliability of the services provided by the organisation as most significant in moderating their experience and loyalty. The survey research indicates that buyers’ psychological factors moderate the relationship between customer experience management and loyalty with customers’ perception as the highest predictor.

## **4.2.11: CONSUMERS’ PERCEPTION OF RESEARCH OBJECTIVES BASED ON THE HOSPITAL OF THE RESPONDENT (SUMMARY OF MEAN)**

**4.2.11a: The Role of Functional Clues in Eliciting Repeat Purchase Actions from Customer of Healthcare Service Firm**

Functional clues have been described as the technical quality of the healthcare offering, revealing the reliability and competence of the service (Berry *et al.*, 2006). In order to ascertain the role of functional clues on repeat purchase actions, this research study specify the first objective of examining the role of functional clues on repeat purchase actions, that is, *“to determine the role of functional clues in eliciting repeat purchase actions from customer of healthcare service firms”.*

In order to actualize this objective, the study raised the first research question which can be restated as follows:

Research Question 1: *“What role do functional clues play in eliciting repeat purchase actions from customers in healthcare service firms”.* In order to obtain answers this question, responses were requested from the healthcare customers and experts.

**Table 4.2.11a: Role of functional clues on repeat purchase**

| S/N | Statements | LGH | RDTH | EKOH | STNH |
| --- | --- | --- | --- | --- | --- |
|  |  | Mean Score | Mean  Score | Mean  Score | Mean Score |
| 1 | The reliability of the health care services. | 3.93 | 4.38 | 3.66 | 4.17 |
| 2 | The competence of the health care service. | 4.24 | 4.49 | 3.73 | 4.13 |
| 3 | The right diagnosis of the health care organisation. | 4.12 | 4.18 | 3.89 | 4.14 |
| 4 | Administration of the right treatment by the health care organisation. | 4.09 | 4.31 | 3.66 | 4.14 |
| 5 | The assured service of the health care organisation. | 4.11 | 4.26 | 3.72 | 4.06 |
| 6 | The procedures of the health care services. | 3.78 | 4.20 | 3.51 | 3.89 |
| 7 | The reliability of the continuous services. | 3.99 | 4.36 | 3.67 | 4.03 |
| 8 | The efficacy of the health care service. | 3.88 | 4.37 | 3.59 | 4.03 |
| 9 | The self-reliance of the health care service organisation. | 4.11 | 4.22 | 3.53 | 4.03 |
| 10 | The practicality of the healthcare services. | 3.91 | 4.09 | 3.52 | 4.01 |

Source: Field Survey, 2016

KEY: LGH = Lagoon Hospital; RDTH= Reddington Hospital,

EKOH = EKO Hospital; STNH = St. Nicholas Hospital

The analysis of the customers’ responses on the role of functional clues on repeat purchase actions reveals positive responses concerning all the statements related to the roles of functional clues on repeat purchase actions as all the mean scores are more than the 3.01 on a 5-linkert point scale. The findings on the above table show that the customer of all the hospitals gave positive responses to the statement. Based on the reliability of the health care services, the mean score range from 3.93 for Lagoon hospital, 4.38 for Reddington Hospital, 3.66 for EKO Hospital and 4.17 for St. Nicholas Hospital. Although they all have a positive response, however, Reddington Hospital has the highest mean score of 4.38, followed by St. Nicholas hospital, this means that the respondents of Reddington and St. Nicholas highly consider the reliability of the healthcare services received from the organisation as the bases for their repeat purchase actions. The survey research finding is in harmony with prior study carried out by Donnelly *et al.* (2008), who revealed that functional clues has a significant association with customer loyalty in the tourism industry. The current state of healthcare services in Nigeria entails that conscious and plan efforts be established to maintain the role which functional clues play in enhancing repeat patronage of the healthcare organisation.

**Table 4.2.11b: Influence of mechanic clues on brand insistence**

| S/N | Statements | LGH | RDTH | EKOH | STNH |
| --- | --- | --- | --- | --- | --- |
|  |  | Mean Score | Mean  Score | Mean  Score | Mean Score |
| 2 | The conducive ambience of the health care organisation | 3.98 | 4.01 | 3.88 | 4.01 |
| 3 | The furniture and fitting of the health care organisation | 3.59 | 3.82 | 3.43 | 3.75 |
| 4 | The facilities of the health care organisation. | 4.09 | 4.16 | 3.93 | 4.02 |
| 5 | The lighting of the health care organisation. | 4.11 | 3.94 | 4.00 | 3.88 |
| 6 | The cleanliness of the health care organisation. | 4.27 | 4.48 | 4.00 | 4.24 |
| 7 | The comfort of the health care lobby/reception | 3.99 | 4.21 | 3.72 | 4.14 |
| 8 | The convenient location of the health care organisation. | 3.91 | 4.15 | 3.84 | 3.94 |
| 9 | The aroma of the health care organisation | 3.38 | 3.85 | 3.42 | 3.22 |

Source: Field Survey, 2016

KEY: LGH = Lagoon Hospital; RDTH= Reddington Hospital,

EKOH = EKO Hospital; STNH = St. Nicholas Hospital

The Table 4.2.11b was designed to measure respondents’ opinion on the effect of mechanic clues on brand insistence based on the hospital of the respondents. From the result presented in Table 4.2.11b, it is evident that all the sampled respondents from the four hospitals gave positive responses concerning all the statements related to the effect of mechanic clues on brand insistence as all the mean scores are more than the 3.01 on a 5-linkert point scale. As clearly seen from the table above, every of the mean scores is on the high side of the measure signifying that, on the average, the respondents viewed the cleanliness, conducive ambience, the physical appearance of the healthcare organisation, furnishings, building design, displays, equipment, colours, smells, sounds, and lighting during their stay in the hospital very positive.

**TABLE 4.2.11c: Influence of humanic clues on switching restraint**

| S/N | Statements | LGH | RDTH | EKOH | STNH |
| --- | --- | --- | --- | --- | --- |
|  |  | Mean Score | Mean  Score | Mean  Score | Mean Score |
| 2 | The caring expression of the health care service providers. | 3.98 | 4.26 | 3.83 | 4.23 |
| 3 | The tone of voice of the health care service provider pleases me. | 4.33 | 4.19 | 3.68 | 3.73 |
| 4 | The body language of the health care service provider. | 3.66 | 4.01 | 3.54 | 3.77 |
| 5 | The respect and courtesy from the health care service provider. | 4.20 | 4.24 | 3.72 | 4.16 |
| 6 | The neatness of the health care service provider fascinates me. | 4.13 | 4.27 | 3.92 | 4.25 |
| 7 | The responsiveness of the health care service provider. | 3.97 | 4.03 | 3.57 | 3.99 |
| 8 | The mindfulness of the health care service provider. | 4.07 | 4.20 | 3.64 | 4.04 |
| 9 | The understanding of the health care service provider. | 4.12 | 4.22 | 3.75 | 4.13 |

Source: Field Survey, 2016

KEY: LGH = Lagoon Hospital; RDTH= Reddington Hospital,

EKOH = EKO Hospital; STNH = St. Nicholas Hospital

Result in Table 4.2.11c underscores how humanic clues influence switching restraint based on the hospital of the respondents. From the result presented in Table 4.2.11c, it is evident that the respondents have positive responses concerning all the statements related to the influence of humanic clues on switching restraint as all the mean scores are more than 3.01 on a 5-linkert point scale. These findings show that the customers of the organisation restrain switching to another organisation as a result of the actions, behaviour and expressions of the employees of the health care organisation in all the hospitals.

**TABLE 4.2.11d: Relationship between customer experience and customer satisfaction**

**in the health care sector**

| S/N | Statements | LGH | RDTH | EKOH | STNH |
| --- | --- | --- | --- | --- | --- |
|  |  | Mean Score | Mean  Score | Mean  Score | Mean Score |
| 2 | The right diagnosis of illness by the health care organisation makes me completely happy with the organisation. | 4.03 | 4.42 | 3.83 | 4.05 |
| 3 | The right treatment of illness by the health care organisation enhances my relief. | 4.18 | 4.39 | 3.83 | 4.27 |
| 4 | The effective verbal communication of the health care service enhances my gratification. | 4.01 | 4.26 | 3.83 | 4.14 |
| 5 | The non-verbal communication of the health care service enhances my gratification. | 3.60 | 3.88 | 3.50 | 3.67 |
| 6 | Respecting my wishes makes me satisfied. | 3.97 | 4.21 | 3.60 | 4.09 |
| 7 | Showing professionally appropriate behaviour by the health care service provider delights me | 4.09 | 4.36 | 3.92 | 4.31 |
| 8 | Maintaining patient privacy enhances customer’s trust. | 4.14 | 4.30 | 4.00 | 4.23 |
| 9 | The aroma of the health care organisation makes me satisfied | 3.64 | 4.04 | 3.70 | 3.65 |
| 10 | The cleanliness of the health care organisation satisfies me | 4.01 | 4.35 | 3.88 | 4.16 |

Source: Field Survey, 2016

KEY: LGH = Lagoon Hospital; RDTH= Reddington Hospital,

EKOH = EKO Hospital; STNH = St. Nicholas Hospital

The above table 4.2.11d was designed to find out the relationship between customer experience and customer satisfaction based on the hospital of the respondents. From the result revealed and presented on the above table, it is evident that all the sampled respondents have a similar response pattern of the relationship between customer experience and customer satisfaction. The table also revealed positive responses concerning all the statements related to the relationship between customer experience and customer satisfaction as all the mean scores are more than 3.01 on a 5-linkert point scale. These findings indicate that the reliability of the healthcare service delivery has a straight relationship with customer satisfaction showing the highest mean score of 4.44 out of all the statement and in all the hospitals. Therefore, the relationship between customer experience and customer satisfaction is direct..

**TABLE 4.2.11e: How buyers’ psychological factors moderate the relationship**

**between CEM & customer loyalty.**

| S/N | Statements | LGH | RDTH | EKOH | STNH |
| --- | --- | --- | --- | --- | --- |
|  |  | Mean Score | Mean  Score | Mean  Score | Mean Score |
| 2 | My interpretation of the right diagnosis of illness. | 4.16 | 4.31 | 3.72 | 4.06 |
| 3 | My motivation about the quality of health care services. | 4.08 | 4.30 | 3.82 | 4.14 |
| 4 | My experience with the behaviour of the health care service provider. | 4.05 | 4.27 | 3.80 | 4.04 |
| 5 | The belief I have concerning the right treatment of illness provided by the organisation. | 4.02 | 4.29 | 3.82 | 4.01 |
| 6 | The maintenance of patient privacy by the healthcare organisation. | 3.97 | 4.18 | 3.95 | 3.92 |
| 7 | The aroma/odor of the healthcare organisation. | 3.51 | 3.90 | 3.65 | 3.66 |
| 8 | My view about the effective verbal communication of the healthcare provider. | 3.95 | 4.24 | 3.78 | 4.10 |
| 9 | My knowledge about the cleanliness of heath care organisation surroundings. | 4.04 | 4.24 | 3.91 | 4.09 |
| 10 | My interpretation of the tone of voice of the health care service provider. | 3.87 | 4.11 | 3.77 | 3.70 |
| 11 | My personality determines how I view the health care services provided. | 4.18 | 4.28 | 3.86 | 4.21 |

Source: Field Survey, 2016

KEY: LGH = Lagoon Hospital; RDTH= Reddington Hospital,

EKOH = EKO Hospital; STNH = St. Nicholas Hospital

The above Table 4.2.11e was designed to find out how buyers’ psychological factors moderate the relationship between customer experience management and customer loyalty based on the hospital of the respondents. From the findings on the above table, it is obvious that all the sampled respondents have similar response pattern. The Table also revealed positive responses concerning all the statements related to all to the research objective as the mean scores are more than 3.01 on a 5-linkert point scale. The findings show that buyers’ psychological factors moderate the relationship between customer experience management and customer loyalty in all the hospitals. It must be noted here that despite the fact that all other variable moderate the relationship between customer experience management and loyalty. Perception serve as the strongest predictor of the relationship because it has the highest mean score of 4.35 compare to all other statements.

## **4.2.12: CONSUMERS’ PERCEPTION OF RESEARCH OBJECTIVES BASED ON PATRONAGE EXPERIENCE (SUMMARY OF MEAN)**

| S/N | Statements | 1-5yrs | 6-10yrs | 11-15yrs | 16yrs+ |
| --- | --- | --- | --- | --- | --- |
|  |  | Mean Score | Mean Score | Mean Score | Mean Score |
| 1 | The reliability of the health care services. | 4.01 | 4.19 | 3.64 | 3.94 |
| 2 | The competence of the health care service. | 4.12 | 4.18 | 4.18 | 4.24 |
| 3 | The right diagnosis of the health care organisation. | 4.08 | 4.13 | 3.91 | 4.00 |
| 4 | Administration of the right treatment by the health care organisation. | 4.02 | 4.09 | 4.09 | 4.12 |
| 5 | The assured service of the health care organisation. | 4.02 | 4.02 | 4.23 | 4.12 |
| 6 | The procedures of the health care services. | 3.78 | 4.00 | 3.77 | 3.82 |
| 7 | The reliability of the continuous services. | 4.05 | 4.03 | 3.68 | 3.82 |
| 8 | The efficacy of the health care service. | 3.89 | 4.11 | 3.82 | 4.24 |
| 9 | The self-reliance of the health care service organisation. | 3.88 | 4.11 | 4.09 | 4.12 |
| 10 | The practicality of the healthcare services. | 3.79 | 4.08 | 3.86 | 3.88 |

**TABLE 4.2.12a: Roles of functional clues on repeat purchase**

Source: Field Survey, 2016

Based on the table 4.2.12a, it is clear that the analysis of customers’ responses on the role of functional clues on repeat purchase actions based on patronage experience of the respondents revealed positive responses concerning all the statements related to the roles of functional clues on repeat purchase actions as all the mean scores are more than the 3.01 on a 5-linkert point scale. The findings on the Table showed that the customer of all the hospitals gave positive responses to all the statement but with special reference to the competence of the healthcare and the efficacy of the healthcare services which gave the highest mean score of 4.24 for 16 and above patronage experience, followed by the assured services of the healthcare organisation which gave a mean score of 4.23 for 11-15 years patronage experience. This findings pointed out that those customers with more patronage experience responded in similar way to the role of functional clues on repeat purchase.

**TABLE 4.2.12b: Influence of mechanic clues on brand insistence**

| S/N | Statements | 1-5yrs | 6-10yrs | 11-15yrs | 16yrs+ |
| --- | --- | --- | --- | --- | --- |
|  |  | Mean Score | Mean Score | Mean Score | Mean Score |
| 1 | The physical appearance of the health care organisation | 4.06 | 4.07 | 3.95 | 4.00 |
| 2 | The conducive ambience of the health care organisation | 4.02 | 3.86 | 3.95 | 4.06 |
| 3 | The furniture and fitting of the health care organisation | 3.73 | 3.57 | 3.50 | 3.29 |
| 4 | The facilities of the health care organisation. | 4.05 | 4.11 | 3.77 | 4.00 |
| 5 | The lighting of the health care organisation. | 3.94 | 3.90 | 4.82 | 4.00 |
| 6 | The cleanliness of the health care organisation. | 4.25 | 4.25 | 3.95 | 4.47 |
| 7 | The comfort of the health care lobby/reception area. | 4.01 | 4.08 | 3.59 | 4.18 |
| 8 | The convenient location of the health care organisation. | 3.95 | 4.01 | 3.82 | 3.94 |
| 9 | The aroma of the health care organisation | 3.53 | 3.42 | 3.36 | 3.06 |

Source: Field Survey, 2016

The above Table 4.2.12b was designed to measure respondents’ opinion on the effect on mechanic clues on brand insistence based on patronage experience. From the result revealed and presented in the above table, it is evident that the patronage experience of the rsepondents have similar response pattern on the effect of mechanic clues on brand insistence. The Table also reveals positive responses concerning all the statements related to the effect of mechanic clues on brand insistence as all the mean scores are more than the 3.01 on a 5-linkert point scale. These findings indicated that the lighting of the healthcare organisation should be focused upon as it gave the highest mean score of 4.82 based on the respondents that have been with the organisation for 11-15 years, followed by the cleanliness of the healthcare organisation with a mean score of 4.47 from the respondents that have been with the organisation for 16 years and above.

**TABLE 4.2.12c: Influences of humanic clues on switching restraint**

| S/N | Responses | 1-5yrs | 6-10yrs | 11-15yrs | 16yrs+ |
| --- | --- | --- | --- | --- | --- |
|  |  | Mean Score | Mean Score | Mean Score | Mean Score |
| 1 | The friendly actions of the health care service providers. | 4.15 | 4.15 | 4.27 | 3.88 |
| 2 | The caring expression of the health care service providers. | 4.08 | 4.06 | 4.09 | 4.06 |
| 3 | The tone of voice of the health care service provider pleases me. | 4.07 | 3.81 | 3.95 | 3.88 |
| 4 | The body language of the health care service provider. | 3.73 | 3.70 | 4.09 | 3.76 |
| 5 | The respect and courtesy from the health care service provider. | 4.01 | 4.18 | 4.09 | 4.29 |
| 6 | The neatness of the health care service provider fascinates me. | 4.11 | 4.23 | 4.09 | 4.12 |
| 7 | The responsiveness of the health care service provider. | 3.87 | 4.01 | 3.50 | 3.82 |
| 8 | The mindfulness of the health care service provider. | 4.00 | 4.00 | 3.73 | 4.00 |
| 9 | The understanding of the health care service provider. | 4.02 | 4.16 | 3.95 | 4.00 |

Source: Field Survey, 2016

Result in Table 4.2.12c highlighted how humanic clues influence switching restraint based on the patronage experience of the respondents. From the result revealed and shown in the Table, it is evident that all the respondents have similar response pattern on the influence of humanic clues on switching restraint. The Table also revealed positive responses concerning all the statements related to the influence of humanic clues on switching restraint as all the mean scores are more than the 3.01 on a 5-linkert point scale. Findings from the Table 4.2.12c concerning those customers that have patronized the hospital for 16 years above revealed that healthcare service provider should have respect and courtesy for their customers as it revealed a mean score of 4.29, followed by the view of those customers that have patronized the hospital for 11 to 15 years, which state that the health care service providers should be friendly as it revealed a mean scores of 4.27.

**TABLE 4.2.12d: Relationship between customer experience and customer satisfaction**

**in the health care sector**

| S/N | Statements | 1-5yrs | 6-10yrs | 11-15yrs | 16yrs+ |
| --- | --- | --- | --- | --- | --- |
|  |  | Mean Score | Mean Score | Mean Score | Mean Score |
| 1 | The reliability of the health care services makes me contented. | 4.16 | 4.15 | 3.82 | 3.88 |
| 2 | The right diagnosis of illness by the health care organisation makes me completely happy with the organisation. | 4.07 | 4.12 | 3.95 | 4.12 |
| 3 | The right treatment of illness by the health care organisation enhances my relief. | 4.20 | 4.17 | 4.00 | 3.82 |
| 4 | The effective verbal communication of the health care service enhances my gratification. | 4.07 | 4.04 | 4.00 | 4.06 |
| 5 | The non-verbal communication of the health care service enhances my gratification. | 3.74 | 3.49 | 3.36 | 4.12 |
| 6 | Respecting my wishes makes me satisfied. | 3.99 | 3.97 | 3.82 | 3.82 |
| 7 | Showing professionally appropriate behaviour by the health care service provider delights me | 4.18 | 4.19 | 3.86 | 4.35 |
| 8 | Maintaining patient privacy enhances customer’s trust. | 4.15 | 4.29 | 4.00 | 3.88 |
| 9 | The aroma of the health care organisation makes me satisfied | 3.81 | 3.70 | 3.68 | 3.47 |
| 10 | The cleanliness of the health care organisation satisfies me | 4.08 | 4.15 | 3.95 | 4.24 |

Source: Field Survey, 2016

The above Table 4.2.12d was designed to investigate the relationship between customer experience and customer satisfaction based on patronage experience of the respondents. Going by the result revealed, it is evident that all the respondents have similar response pattern on the relationship between customer experience and customer satisfaction. The Table also revealed positive responses concerning all the statements related to the relationship between customer experience and customer satisfaction as all the mean scores are more than the 3.01 on a 5-linkert point scale. These findings showed that those customers that have patronized the hospital for 6-10 years are of the opinion that maintaining patient privacy enhances their customers’ trust, followed by those that have patronized the hospital for 16 years and above stating that the cleanliness of the healthcare service organisation lead to satisfaction.

**TABLE 4.2.12e: How buyers’ psychological factors moderate the relationship between**

**CEM & customer loyalty.**

| S/N | Statements | 1-5yrs  Mean Score | 6-10yrs  Mean Score | 11-15yrs  Mean Score | 16yrs+  Mean Score |
| --- | --- | --- | --- | --- | --- |
| 1 | My perception about the reliability of services provided | 4.19 | 4.18 | 4.05 | 4.00 |
| 2 | My interpretation of the right diagnosis of illness. | 4.03 | 4.13 | 4.18 | 3.88 |
| 3 | My motivation about the quality of health care services. | 4.09 | 4.06 | 4.09 | 4.18 |
| 4 | My experience with the behaviour of the health care service provider. | 4.03 | 4.08 | 3.91 | 4.12 |
| 5 | The belief I have concerning the right treatment of illness provided | 4.03 | 4.04 | 4.05 | 4.00 |
| 6 | The maintenance of patient privacy by the healthcare organisation. | 3.97 | 4.12 | 3.77 | 3.94 |
| 7 | The aroma/odor of the healthcare organisation. | 3.74 | 3.62 | 3.64 | 3.24 |
| 8 | My view about the effective verbal communication of the healthcare provider | 4.02 | 4.07 | 3.91 | 3.76 |
| 9 | My knowledge about the cleanliness of heath care organisation surroundings. | 4.08 | 4.05 | 4.00 | 4.12 |
| 10 | My interpretation of the tone of voice of the health care service provider. | 3.85 | 3.88 | 3.82 | 3.88 |
| 11 | My personality determines how I view the health care services provided. | 4.12 | 4.18 | 4.05 | 4.00 |

Source: Field Survey, 2016

The Table 4.2.12e was designed to find out how the buyers’ psychological factors moderate the relationship between customer experience management and customer loyalty based on patronage experience of the respondents. This Table revealed that the consumer perception about the reliability of healthcare services provided for those respondents that have patronized the hospital for 1-5 years have the highest mean score of 4.19, this was followed by the motivation of customer with a mean score of 4.18. Given the totality of the result, it is an indication that the patronage experience of the respondents agreed or strongly agreed with all the variables concerning how buyers’ psychological factors moderate the relationship between customer experience management and customer loyalty.

**4.2.13 VARIATION IN PERCEPTION ACROSS DEMOGRAPHIC CHARCTERISTCS OF RESPONDENTS BASED ON THE OBJECTIVES**

**Table 4.2.13a: Variation in Perception of the Customers on the Role of Functional Clues on Repeat Purchase by the Respondents**

| **Test Statistics^a,b^** | | | | | | | | |
| --- | --- | --- | --- | --- | --- | --- | --- | --- |
|  | Gender | Age | Educational Qualification | Marital status | Employment status | Class of customers | Hospitals patronized | Years of Patronage Experience |
| Chi-Square | 12.351 | 13.149 | 14.299 | 10.710 | 6.009 | 5.080 | 5.267 | 11.102 |
| Df | 4 | 4 | 4 | 4 | 4 | 4 | 4 | 4 |
| Asymp. Sig. | .015 | .011 | .006 | .030 | .198 | .279 | .261 | .025 |
| a. Kruskal Wallis Test | | | | | | | | |
| b. Grouping Variable: Repeat purchase | | | | | | | | |

Using repeat purchase as the dependent variable and the personal profiles (attribute) of the respondents (gender, age, educational qualification, marital status, employment status, class of customers, hospitals patronized and patronage experience of the customer) as the independent variables, Kruskal Wallis Test was carried out to examine factors responsible for the observed result on the role of functional clues on repeat purchase. The test showed that difference in the perception of the customers on the role of functional clues on repeat purchase by employment status (λ^2^ = 6.009, df=4, P>0.05), class of customers (λ^2^ = 5.080, df=4, P>0.05), hospital patronized (λ^2^ = 5.267, df=4, P>0.05) are not statistically significant. This means that the difference in the perception of the customers on the role of functional clues on repeat purchase actions are not due to employment status, class of customers and the hospital patronized by the customers. The result also revealed that the variance in the opinion of the consumers on the role of functional clues on repeat purchase by gender (λ^2^ = 12.351, df=4, P<0.05), age (λ^2^ = 13.149, df=4, P<0.05), educational background (λ^2^ = 14.299, df=4, P<0.05), marital status (λ^2^ = 10.710, df=4, P<0.05), and patronage experience (λ^2^ = 11.102, df=4, P<0.05) are statistically significant. This implies that the differences in the perception of the customer on the role of functional clues on repeat purchase can be linked to differences in gender, age, educational qualifications and years of patronage experience of the respondents.

**Table 4.2.13b: Variation in Perception of the Customers on the Influence of Mechanic Clues on Brand Insistence by the Respondents**

| **Test Statistics^a,b^** | | | | | | | | |
| --- | --- | --- | --- | --- | --- | --- | --- | --- |
|  | Gender | Age | Educational Qualification | Marital status | Employment status | Class of customers | Hospitals patronized | Years of Patronage Experience |
| Chi-Square | 3.567 | 8.667 | 10.208 | 7.090 | 8.188 | 4.058 | 1.250 | 2.753 |
| Df | 4 | 4 | 4 | 4 | 4 | 4 | 4 | 4 |
| Asymp. Sig. | .468 | .070 | .037 | .131 | .085 | .398 | .870 | .600 |
| a. Kruskal Wallis Test | | | | | | | | |
| - 1. b. Grouping Variable: Brand insistence   Using brand insistence as the dependent variable and the personal profile (attributes) of the respondents (gender, age, educational qualification, marital status, employment status, class of customers, hospitals patronized and years of patronage experience of the customer) as the independent variables. Kruskal Wallis Test was carried out to investigate and identify what accounted for the variation in the customers’ perception of the influence of mechanic clues on brand insistence. The test showed that difference in the perception of customer on the effect of mechanic clues on brand insistence by gender (λ^2^ = 3.567, df=4, P>0.05), age (λ^2^ = 8.667, df=4, P>0.05), marital status (λ^2^ = 7.090, df=4, P>0.05), employment status (λ^2^ = 8.188, df=4, P>0.05), class of customers (λ^2^ = 4.058, df=4, P>0.05), hospital patronized (λ^2^ = 1.250, df=4, P>0.05) and years of patronage experience (λ^2^ = 2.753, df=4, P>0.05) are not statistically significant. This means that the variance in the opinion of consumers on the influence of mechanic clues on brand insistence are not due to the variation in gender, age, marital status, employment status, class of customers, hospitals patronized and years of patronage experience of the customers. However, the result also revealed that the difference in the perception of the customers on the influence of mechanic clues on brand insistence by educational qualification (λ^2^ = 10.208, df=4, P<0.05) is statistically significant. This implies that the differences in the perception of the customers on the influence of mechanic clues on brand insistence can be linked to the different levels of educational qualifications of the respondents that participated in the surveys. | | | | | | | | |

**Table 4.2.13c: Variation in Perception of the Customers on the Influence of Humanic Clues on Switching Restraint**

| **Test Statistics^a,b^** | | | | | | | | |
| --- | --- | --- | --- | --- | --- | --- | --- | --- |
|  | Gender | Age | Educational Qualification | Marital status | Employment status | Class of customers | Hospitals patronized | Years of Patronage Experience |
| Chi-Square | 1.315 | 16.391 | 15.856 | 10.986 | 7.673 | 3.259 | 5.623 | 1.488 |
| Df | 4 | 4 | 4 | 4 | 4 | 4 | 4 | 4 |
| Asymp. Sig. | .859 | .003 | .003 | .027 | .104 | .516 | .229 | .829 |
| a. Kruskal Wallis Test | | | | | | | | |
| b. Grouping Variable: Switching restraint | | | | | | | | |

Using switching restraint as the dependent variables and the personal attributes of the respondents (gender, age, educational qualifications, marital status, employment status, class of customers, hospitals patronized and years of patronage experience of the customer) as the independent variables. Kruskal Wallis Test was carried out to investigate differences in the perception of the influence of humanic clues on switching restraints. The test showed that difference in the perception of customer on the influence of humanic clues on switching restraint by gender (λ^2^ = 1.315, df=4, P>0.05), employment status (λ^2^ = 7.673, df=4, P>0.05), class of customers (λ^2^ = 3.259, df=4, P>0.05), hospital patronized (λ^2^ = 5.623, df=4, P>0.05) and years of patronage experience (λ^2^ = 1.488, df=4, P>0.05) are not statistically significant. This means that the difference in the perception of customers on the influence of humanic clues on switching restraint of customers are not due to the differences in gender, employment status, class of customers, hospital patronized and the years of patronage experience of the customers. On the other hand, the result revealed that the variance in the opinion of consumers on the influence of humanic clues on switching restraint by age (λ^2^ = 16.391, df=4, P<0.05), educational qualifications (λ^2^ = 15.856, df=4, P<0.05), and marital status (λ^2^ = 10.986, df=4, P<0.05) are statistically significant. This implies that the difference in the perception of the customers on the influence of humanic clues on switching restraint can be linked to the variation in age, educational qualifications and marital status of the customer.

**Table 4.2.13d: Variation in Perception of the Customers on the Relationship between Customer Experience and Satisfaction**

| **Test Statistics^a,b^** | | | | | | | | |
| --- | --- | --- | --- | --- | --- | --- | --- | --- |
|  | Gender | Age | Educational Qualification | Marital status | Employment status | Class of customers | Hospitals patronized | Years of Patronage Experience |
| Chi-Square | 6.165 | 11.007 | 1.308 | 4.832 | 5.889 | 7.416 | 5.892 | 4.144 |
| Df | 4 | 4 | 4 | 4 | 4 | 4 | 4 | 4 |
| Asymp. Sig. | .187 | .026 | .860 | .305 | .208 | .115 | .207 | .387 |
| a. Kruskal Wallis Test | | | | | | | | |
| b. Grouping Variable: Customer satisfaction | | | | | | | | |

Using customer satisfaction as the dependent variable and the personal characteristics of the respondents (gender, age, educational qualifications, marital status, employment status, class of customers, hospitals patronized and years of patronage experience of the customer) as the independent variables. Kruskal Wallis Test was carried out to examine what might have accounted for the variation in perception of the relationship between customer experience and satisfaction with healthcare services in the four hospitals sampled. The test showed that difference in the perception of the customers on the relationship between customer experience and satisfaction by gender (λ^2^ = 6.165, df=4, P>0.05), educational qualifications (λ^2^ = 1.308, df=4, P>0.05), marital status (λ^2^ = 4.832, df=4, P>0.05), employment status (λ^2^ = 5.889, df=4, P>0.05), class of customers (λ^2^ = 7.416, df=4, P>0.05), hospital patronized (λ^2^ = 5.892, df=4, P>0.05) and years of patronage experience (λ^2^ = 4.144, df=4, P>0.05) are not statistically significant. This means that the difference in the perception of customers on the relationship between customer experience and customer satisfaction are not due to differences in gender, educational qualifications, marital status, employment status, class of customers, hospitals patronized and the years of patronage experience of the customer. The result however revealed that the variances in the opinion of the consumers on the relationship between customer experience and customer satisfaction by age (λ^2^ = 11.007, df=4, P<0.05) is statistically significant. This implies that the differences in the perception of customer on the relationship between customer experience and customer satisfaction can be linked to the age differences of the consumers.

**Tables 4.2.13e: Variation in Perception of the Customers on the Moderating role of Buyers’ Psychological Factors in the Relationship between Customer Experience Management and Loyalty**

| **Test Statistics^a,b^** | | | | | | | | |
| --- | --- | --- | --- | --- | --- | --- | --- | --- |
|  | Gender | Age | Educational Qualification | Marital status | Employment status | Class of customers | Hospitals patronized | Years of Patronage Experience |
| Chi-Square | 5.127 | 5.010 | 16.454 | 1.194 | 7.599 | 8.148 | .741 | 4.299 |
| Df | 4 | 4 | 4 | 4 | 4 | 4 | 4 | 4 |
| Asymp. Sig. | .274 | .286 | .002 | .879 | .107 | .086 | .946 | .367 |
| a. Kruskal Wallis Test | | | | | | | | |
| b. Grouping Variable: buyer’s psychological factor | | | | | | | | |

Using buyers’ psychological factors as the dependent variable and the personal characteristics of the respondents (gender, age, educational qualifications, marital status, employment status, class of customers, hospitals patronized and years patronage experience of the customers) as the independent variables. Kruskal Wallis Test was carried out to investigate the factors responsible for the variation in perception on the moderation role of buyers’ psychological factors on the relationship between CEM and loyalty. The test shows that difference in the perception of the customers on the moderating role of buyers’ psychological factors in the relationship between customer experience management and customer loyalty by gender (λ^2^ = 5.127, df=4, P>0.05), age (λ^2^ = 5.010, df=4, P>0.05), marital status (λ^2^ = 1.194, df=4, P>0.05), employment status (λ^2^ = 7.599, df=4, P>0.05), class of customers (λ^2^ =8.148, df=4, P>0.05), hospital patronized (λ^2^ = .741, df=4, P>0.05) and the years of patronage experience (λ^2^ = 4.299, df=4, P>0.05) are not statistically significant. This means that the difference in the perception of customers on the moderating role of buyers’ psychological factors in the relationship between customer experience management and customer loyalty are not due to differences in gender, age, marital status, employment status, class of customers, hospitals patronized and the years of patronage experience of the customers. The result also revealed that the difference in the perception of customer on the moderating role of buyers’ psychological factors in the relationship between customer experience management and customer loyalty by educational background (λ^2^ = 16.454, df=4, P<0.05) is statistically significant. This implies that the differences in the perception of the customers on the moderating role of buyers’ psychological factors in the relationship between customer experience management and customer loyalty can be linked to the differences educational qualifications among the respondents in the survey.

**4.2.14 Analysis of the Responses to Open Ended Questions**

The open ended questions aspect of the questionnaire was analyzed using descriptive statistics. The respondents were asked to indicate other ways healthcare organisations can improve the experience of their customers that will lead to customer loyalty. The result is presented in Table 4.2.14a.

**Table 4.2.14a: How to Improve the Experiences of Customers by Healthcare Organisation that will lead to Customer Loyalty**

| **Ss s/n** | **T Themes** | **Freq Frequency** | **Pe c Percent(%)** | **Ranking** |
| --- | --- | --- | --- | --- |
| 1. | Timely/quick response to customers | 40 | 14.2 | 1 |
| 2. | Good relationship with customers | 32 | 11.3 | 2 |
| 3. | Respect for customers by service provider | 27 | 9.6 | 3 |
| 4. | Ensure delivery of maximum customer satisfaction | 26 | 9.2 | 4 |
| 5. | Consistence cleanliness of the environment | 21 | 7.5 | 5 |
| 6. | Saving life before money should be the healthcare organisation’s priority | 17 | 6.1 | 6 |
| 7. | Patience and listening to customers | 15 | 5.3 | 7 |
| 8. | Adequate quality control measures | 13 | 4.7 | 8 |
| 9. | Creating conducive environment | 11 | 3.9 | 9 |
| 10. | Adequate modern healthcare facilities | 10 | 3.5 | 10 |
| 11. | Private keeping of customer’s information | 9 | 3.2 | 11 |
| 12. | Effective communication | 9 | 3.2 | 11 |
| 13. | Doing it right the first time | 9 | 3.2 | 11 |
| 14. | Reduction of hospital bills | 8 | 2.8 | 12 |
| 15. | Training and retraining of service providers | 8 | 2.8 | 12 |
| 16. | Regular check up on patient via phone calls | 7 | 2.5 | 13 |
| 17. | Introduction of e-healthcare modern equipment | 6 | 2.1 | 14 |
| 18. | Free medicine to children from 1-5 years | 6 | 2.1 | 14 |
| 19. | Avoidance of favouritism in service delivery | 4 | 1.4 | 15 |
| 20. | Provision of dedicated toll free numbers for customers to call | 4 | 1.4 | 15 |

Source: Researcher’s Field Survey, 2016

The result presented in Table 4.2.14a reveals that a majority of the customers 40(14.2%) indicated that timely and quick response to customers can help healthcare organisations to improve the experience of their customers leading to customer loyalty. This is followed by good relationship with customers 32(11.3 %), respect for customers by service provider 27(9.6%), ensuring delivery of maximum customer satisfaction 26(9.2%), and consistency in cleanliness of the environment 21(7.5%).

**4.3 TEST OF HYPOTHESES**

Research hypotheses are seen as answers to the research questions, and as such, they have to be tested to know if they should be accepted or rejected. There are five hypotheses stated and tested in this study. Testing of theses hypotheses and their final interpretation helped the researcher to know what final decision should be taken.

**Test of Hypothesis No 1**

The first hypothesis of this study was formulated based on the research question and objective no 1

**Research Question One (RQ1) is:**

What roles do functional clues play in eliciting repeat purchase actions of customers in healthcare service firms in Nigeria?

**Research Objective One (RO1) is:**

To determine the roles of functional clues in eliciting repeat purchase actions from consumer of healthcare service firms in Nigeria.

**Research Hypothesis One (H_1_) is:**

Functional clues do not play any significant role in eliciting repeat purchase actions of customers in the healthcare service firms in Nigeria.

The categorical regression analysis and analysis of variance were employed to test the hypothesis. This study employed the categorical regression analysis because the data is the combination of ordinal and nominal data. This was used to examine the predictive capabilities of functional clues on repeat purchase actions of customers in the four private hospitals investigated in this research.

**Decision Rule for the Analysis**

The significance level below 0.05 illustrates the confidence level of 95%. Hence, under this condition, we reject the null (H_0_) hypothesis once P-value is less than or equals to 0.05, while we accept the alternate (H_1_) hypothesis.

**Table 4.3.1a** is the regression model summary of the roles of the functional clues in eliciting repeat purchase actions by customers in the healthcare organisations sampled.

From the result as presented in Table 4.3.1a, it is evident that the model having R^2^ = 0.705 explains around 71% of variance in the respondents’ perception of the role of functional clues in eliciting repeat purchase of healthcare services in the four hospitals sampled.

| **TABLE 4.3.1a: Model Summary^a^  of the Role of Functional Clues in Repeat Purchase** | | | | |
| --- | --- | --- | --- | --- |
| Multiple R | R Square | Adjusted R Square | Apparent Prediction Error |  |
| .840 | .705 | .692 | .295 |  |
| 1. Predictors: (Constant), Functional Clues: The assured service ,the reliability of the health care services, the right diagnosis, administration of the right treatment and the competence of the health care service   Dependent variable: Repeat purchase  Source: Researcher’s Field Survey, 2016 | | | | |

| Table 4.3.1b is the ANOVA of the regression analysis of the roles of functional clues on repeat purchase. It is evident from the result (Table 4.3.1b) that the regression model has F (16,348) = 52.029 and P < 0.000. This means that the null hypothesis (H_0_) is rejected since P-value is less than 0.05. Consequently, from Table 4.3.1b the functional clues are statistically significant to repeat purchase actions.  **Table 4.3.1b : ANOVA^b^ of the Role of Functional Clues in Repeat Purchase** | | | | | | |  |
| --- | --- | --- | --- | --- | --- | --- | --- |
| Model | | Sum of Squares | Df | Mean Square | F | Sig. |  |
|  | Regression | 257.398 | 16 | 16.087 | 52.029 | .000 |  |
|  | Residual | 107.602 | 348 | .309 |  |  |  |
|  | Total | 365.000 | 364 |  |  |  |  |
| 1. Predictors: (Constant), Functional clues 2. Dependent Variable: Repeat purchase   **Table 4.3.1c: Regression Coefficients of the Role of Functional Clues in Repeat Purchase** | | | | | | |  |
| \| **Table 4.3.1c: Coefficients** \| \| \| \| \| \| \| --- \| --- \| --- \| --- \| --- \| --- \| \| Independent Variables \| Standardized Coefficients \| \| Df \| F \| Sig. \| \| Beta \| Bootstrap (1000) Estimate of Std. Error \| \| The reliability of the health care services \| .083 \| .113 \| 1 \| .536 \| .465 \| \| The competence of the health care service \| .328 \| .152 \| 4 \| 4.636 \| .001* \| \| The right diagnosis of the health care organisation \| .296 \| .157 \| 3 \| 3.564 \| .014* \| \| Administration of the right treatment by the health care organisation \| .130 \| .109 \| 4 \| 1.418 \| .227 \| \| The assured service of the health care organisation \| .249 \| .143 \| 4 \| 3.018 \| .018* \| \| Dependent Variable: Repeat Purchase \| \| \| \| \| \| | | | | | | | |

* Significant predictors

*Source: Researcher’s Field Survey, 2016*

Examination of the results in Table 4.3.1c revealed that three factors emerged as the significant predictors and contributed most to explaining the role of functional clues in eliciting repeat purchase actions by the customers sampled. These predictors are the competence of the healthcare services providers, the right diagnosis by the healthcare service providers and the assured services of the healthcare organisation. These three predictors have the following p-value (0.001, 0.014 and 0.018, respectively). Table 4.3.1c, also shows that the competence of the healthcare service organisation having the highest beta value of (*β* = .328) contribute most to explaining the role of functional clues in repeat purchase of healthcare services. This is followed by the right diagnosis of the healthcare organisation (*beta* = .296) and the assured service of the healthcare organisation (*beta* = .249) respectively. This means that the competence of the healthcare service makes the strongest unique contribution to explaining repeat purchase actions of customers than the other variables investigated in this research.

The research also investigated which aspects of functional clues predict repeat purchase actions by customers from the perspective of the healthcare experts and managers. This was done by carrying out CATREG analysis and the model summary is shown in Table 4.3.1d. It is evident from Table 4.3.1d that with R^2^ = 0.615 explained around 62% of variance in the experts’ views on the role of functional clues on repeat purchase of healthcare services by the customers.

| **Table 4.3.1d: Model Summary** | | | | |
| --- | --- | --- | --- | --- |
| Multiple R | R Square | Adjusted R Square | Apparent Prediction  Error |  |
| .784 | .615 | .588 | .385 |  |
| Predictors: (Constant), The competence of our health care team, The efficacy of the drugs dispensed to patients, The quality of our health care services, The reliability of our health care | | | | |

Dependent variable: Repeat purchase

*Source: Researcher’s Field Survey, 2016*

The ANOVA of the regression model is also presented in Table 4.3.1e. It is also evident from the result (Table 4.3.1e) that the regression model has F (8,115) = 22.921 is significant with P< 0.005. Again, this means that the null hypothesis (H_0_) is rejected as we accept the alternative hypothesis.

| **Table 4.3.1e: ANOVA^a^** | | | | | | |
| --- | --- | --- | --- | --- | --- | --- |
|  | | Sum of Squares | Df | Mean Square | F | Sig. |
|  | Regression | 76.207 | 8 | 9.526 | 22.921 | .000 |
|  | Residual | 47.793 | 115 | .416 |  |  |
|  | Total | 124.000 | 123 |  |  |  |

1. Predictors: (Constant), Functional clues

Dependent Variable: Repeat purchase

*Source: Researcher’s Field Survey, 2016*

| **Table 4.3.1f: Coefficients of Regression Analysis of the Role of Functional Clues on Repeat Purchase by Health Care Experts** | | | | | | |
| --- | --- | --- | --- | --- | --- | --- |
|  | Standardized Coefficients | | Df | F | Sig. |  |
|  | Beta | Bootstrap (1000) Estimate of Std. Error |  |  |  |  |
| The reliability of our health care facilities | .156 | .153 | 2 | 1.029 | .361 |  |
| The efficacy of the drugs dispensed to patients | .455 | .098 | 3 | 21.388 | .000* |  |
| The quality of our health care services | .261 | .100 | 2 | 6.828 | .002* |  |
| The competence of our health care team. | .307 | .078 | 1 | 15.604 | .000* |  |
| Dependent Variable: Repeatpurchase | | | | | |  |
| * Significant predictors | | | | | |  |

*Source: Researcher’s Field Survey, 2016*

Table 4.3.1f shows the coefficients of the regression analysis of the healthcare experts opinion on the roles of functional clues on repeat purchase by customers of the four hospitals investigated. The result in Table 4.3.1f reveals that three factors, namely: the efficacy of the drugs dispensed to patients, the quality of healthcare services and the competence of the healthcare team emerged as the aspects of functional clues to predict the repeat purchase actions of customers of the healthcare organisations. Further, based on the beta values of those predictors as shown in Table 4.3.1f it is also evident that the efficacy of the drugs dispensed to patients with the highest beta value of (*β* = .455) contributes mostly in explaining repeat purchase actions by customers. This is followed by the competence of the healthcare team (*beta* = .307) and the quality of our health care services (*beta* = .261). This means that healthcare experts in the hospital believe that the efficacy of the drugs dispensed to patients by the healthcare organisation makes the strongest unique contribution in influencing the repeat purchase actions of customers.

## **Test of Hypothesis 2**

The second hypothesis was formulated based on the research question and research objective two of the study

Research Question 2 is:

How does the mechanic clue influence the brand insistence of customers in the health care service sector of Nigeria?

Research Objective 2:

To investigate whether mechanic clues of health care service influence the brand insistence of customers in the health care sector of Nigeria.

Research Hypothesis 2

Mechanic clues do not have significant influence on the brand insistence of customers in the health care sector in Nigeria.

**Decision rule for analysis**

The significance level below 0.05 illustrates the confidence level of 95%. Hence, under this condition the null (H_0_) hypothesis is rejected once P-value is less than or equals to 0.05 while we accept the alternate (H_1_) hypothesis.

In testing this hypothesis from the customers’ perspective CATREG analysis was conducted using brand insistence as the dependent variable and mechanic clues as the independent variables. The result in Table 4.3.2a shows that the model has R^2^ = 0.517, F (16,348) = 23.284 and P < 0.000. This means that model explains around 52% of the variance on the customers’ perception of the influence of mechanic clues on brand insistence in the four private hospitals investigated.

**Table 4.3.2a:** Regression effects of mechanic clues on brand insistence of customers in the healthcare service firms in Nigeria (Customers’ perspective)

| **Table 4.3.2a: Model Summary of Mechanic Clues on Brand Insistence** | | | | |
| --- | --- | --- | --- | --- |
| Multiple R | R Square | Adjusted R Square | Apparent Prediction Error |  |
| .719 | .517 | .495 | .483 |  |
| 1. Predictors: (Constant), Mechanic Clues: The lighting of the health care organisation, the conducive ambience, the facilities of the health care organisation, the furniture and fitting and the physical appearance of the health care organisation. 2. Dependent Variable: Brand Insistence | | | | |

| *Source: Researcher’s Field Survey, 2016* |
| --- |

Table 4.3.2b is the ANOVA of the regression analysis of the influence of mechanic clues on brand insistence. It is evident from the result (Table 4.3.2b) that the regression model has F (16,348) = 23.284 and P < 0.000. This means that the null hypothesis (H_0_) is rejected since P-value is less than 0.05. Consequently, from Table 4.3.2b the mechanic clues are statistically significant to brand insistence.

| **Table 4.3.2b: ANOVA of Mechanic Clues on Brand Insistence** | | | | | | | |
| --- | --- | --- | --- | --- | --- | --- | --- |
| Model | | Sum of Squares | Df | Mean Square | F | Sig. | |
| 1 | Regression | 188.718 | 16 | 11.795 | 23.284 | .000 | |
|  | Residual | 176.282 | 348 | .507 |  |  | |
|  | Total | 365.000 | 364 |  |  |  | |
| 1. Predictors: (Constant), Mechanic Clues 2. Dependent Variable: Brand Insistence | | | | | | | |

*Source: Researcher’s Field Survey, 2016*

| \|  \| \| --- \|  \| **Table 4.3.2c: Coefficients of Regression Analysis of the Influence of Mechanic Clues on Brand Insistence of healthcare customers** \| \| \| \| \| \| \| --- \| --- \| --- \| --- \| --- \| --- \| \|  \| Standardized Coefficients \| \| Df \| F \| Sig. \| \| Beta \| Bootstrap (1000) Estimate of Std. Error \| \| The physical appearance of the health care organisation. \| .180 \| .095 \| 3 \| 3.605 \| .014* \| \| The conducive ambience of the health care organisation \| .242 \| .239 \| 3 \| 1.026 \| .381 \| \| The furniture and fitting of the health care organisation \| .251 \| .117 \| 3 \| 4.655 \| .003* \| \| The facilities of the health care organisation. \| .216 \| .121 \| 4 \| 3.167 \| .014* \| \| The lighting of the health care organisation. \| .207 \| .104 \| 3 \| 3.999 \| .008* \| \| 1. Dependent Variable: Brand insistence \| \| \| \| \| \| \| * Significant predictors  *Source: Researcher’s Field Survey, 2016* \| \| \| \| \| \|   The model in Table 4.3.2c, revealed that the furniture and fitting of the health care organisation had more statistical significance in predicting brand insistence of customers, recording the highest beta value of (β = .251) contributes most in explaining brand insistence by customers. This is followed by the facilities of the health care organisation (*beta* = .216), the physical appearance of the health care organisation (*beta* = .180) and the lighting of the health care organisation (*beta* = .207). This means that the customers of the healthcare organisations believe that furniture and fitting of the health care organisation makes the strongest unique contribution in explaining brand insistence. | | | | | | | |
| --- | --- | --- | --- | --- | --- | --- | --- | --- | --- | --- | --- | --- | --- | --- | --- | --- | --- | --- | --- | --- | --- | --- | --- | --- | --- | --- | --- | --- | --- | --- | --- | --- | --- | --- | --- | --- | --- | --- | --- | --- | --- | --- | --- | --- | --- | --- | --- | --- | --- | --- | --- | --- | --- | --- | --- | --- | --- | --- | --- | --- | --- | --- | --- | --- |
| **Table 4.3.2: Regression effects of mechanic clues on brand insistence of customers in the healthcare service firms in Nigeria (Healthcare Experts’ Perspective)**  Table 4.3.2d shows the combined influence of the independent variables (the adequacy of our health care facilities, the physical esthetics of our health care facility, the furniture and fitting of the healthcare organisation, the conducive ambience of our health care organisation.) on brand insistence (the dependent variable) of healthcare service customers. Based on the result, the mechanic clues made significant joint influence on the brand insistence of healthcare service customers (R=0.926). Also, the Table gives the R^2^value to be 0.858 indicating that the total contribution or influence made by all the independent variables was 85.8%. This signifies that the mechanic clues had significant joint influence on the brand insistence of healthcare service customers.   \| **Table 4.3.2d: Model Summary** \| \| \| \| \| \| --- \| --- \| --- \| --- \| --- \| \| Multiple R \| R Square \| Adjusted R Square \| Apparent Prediction Error \| \| .926 \| .858 \| .845 \| .142 \| \| a. Predictors: (Constant), The adequacy of our health care facilities The physical esthetics of our health care facility, The furniture and fitting of the healthcare organisation, The conducive ambience of our health care organisation. \| \| \| \| \|  1. Dependent Variable: Brand Insistence   *Source: Researcher’s Field Survey, 2016* | | | | | | | |
| The result in the Table 4.3.2e establishes that the composite influence of the functional clues did not occur by chance as it gives the F-ratio value of 68.092 which signifies the strength of the three independent variables (under the mechanic clues) as potent predictors of brand insistence of healthcare service customers.   \| **Table 4.3.2e:ANOVA** \| \| \| \| \| \| \| --- \| --- \| --- \| --- \| --- \| --- \| \|  \| Sum of Squares \| Df \| Mean Square \| F \| Sig. \| \| Regression \| 106.351 \| 10 \| 10.635 \| 68.092 \| .000 \| \| Residual \| 17.649 \| 113 \| .156 \|  \|  \| \| Total \| 124.000 \| 123 \|  \|  \|  \|  1. Predictors: (Constant), Mechanic Clues 2. Dependent Variable: Brand Insistence   *Source: Researcher’s Field Survey, 2016* | | | | | |  |  |
|  | | | | | |  |  |
| **Table 4.3.2f: Coefficients** | | | | | | |  |
|  | Standardized Coefficients | | Df | F | Sig. | |  |
|  | Beta | Bootstrap (1000) Estimate of Std. Error |  |  |  |  |  |
| The physical esthetics of our health care facility. | -.226 | .186 | 2 | 1.480 | .232 | |  |
| The conducive ambience of our health care organisation. | .672 | .290 | 3 | 5.379 | .002* | |  |
| The furniture and fitting of our health care organisation | .694 | .217 | 3 | 1.840 | .001* | |  |
| The adequacy of our health care facilities. | .210 | .252 | 2 | .697 | .500 | |  |
| Dependent Variable: Brand Insistence | | | | | | |  |
| * Significant predictors  *Source: Researcher’s Field Survey, 2016* | | | | | | |  |

The result in the Table 4.3.2f shows the healthcare experts opinion on the influence of mechanic clues on brand insistence and it reveals that the furniture and fitting of the health care organisation had more statistical significance in predicting brand insistence of customers, recording the highest beta value of (*β* = .694) contributes mostly in explaining the influence of mechanic clues on brand insistence of customers. This is followed by the conducive ambience of the health care organisation (*beta* = .674). This means that the furniture and fitting of the health care organisation makes the strongest unique contribution in explaining brand insistence of customers.

## **Test of Hypothesis 3**

The third hypothesis was formulated based on research question and research objective

Research Question 3

In what ways do humanic clues affect customers switching restraint in health care sector of Nigeria?

Research Objective 3

To examine the extent to which the humanic clues affect the switching restraint of customers in the health care sector of Nigeria.

Research Hypothesis 3

Humanic clues do not have significant effect on switching restraints of customers in the health care sector of Nigeria.

**Decision rule for analysis**

The significance level below 0.05 illustrates the confidence level of 95%. Hence, under this condition the null (H_0_) hypothesis is rejected once P-value is less than or equals to 0.05 while we accept the alternate (H_1_) hypothesis.

**Table 4.3.3a:** Is the regression model summary of the role of the humanic clues on switching restraint of customers in the healthcare organisation sampled.

From the result presented in Table 4.3.3a, the R Square tells how much of the variance in the dependent variable (Switching restraint) is explained by the model (Humanic Clues: the respect and courtesy, tone of voice, friendly actions, body language, caring expression). Here, the R Square value of .994 is stated as a percentage; this implies that the model (humanic clues) elucidates 99.4% of the variance on brand insistence. This is very reasonable result when compare to some outcome that are reported in the journals

| \| **Table 4.3.3a: Model Summary of the Influence of Humanic Clues on Switching Restraint** \| \| \| \| \| \| --- \| --- \| --- \| --- \| --- \| \| Multiple R \| R Square \| Adjusted R Square \| Apparent Prediction Error \| \| .997 \| .994^a^ \| .993 \| .006 \| \| 1. Predictors: (Constant), Humanic clues: the respect and courtesy, tone of voice, friendly actions, body language, caring expression 2. Dependent variable: Switching Restraint   *Source: Researcher’s Field Survey, 2016* \| \| \| \| \|  \|  \| \| --- \|   The Table 4.3.3b shows the ANOVA of the regression analysis of the influence of humanic clues on switching restraints. It is evident from the result (Table 4.3.3b) that the regression model have F (19, 345) = 2852.966 and P < 0.000. This means that the null hypothesis (H_0_) is rejected since P-value is less than 0.05. Consequently, from Table 4.3.3b the humanic clues are statistically significant to switching restraint. |
| --- | --- | --- | --- | --- | --- | --- | --- | --- | --- | --- | --- | --- | --- | --- | --- | --- | --- | --- | --- |

| **Table 4.4.3b: ANOVA^a^ of the Influence of Humanic Clues on Switching Restraint** | | | | | | | |
| --- | --- | --- | --- | --- | --- | --- | --- |
|  | | Sum of Squares | Df | Mean Square | F | Sig. |  |
|  | Regression | 362.692 | 19 | 19.089 | 2852.966 | .000 |  |
|  | Residual | 2.308 | 345 | .007 |  |  |  |
|  | Total | 365.000 | 364 |  |  |  |  |

| **Table 4.3.3c: Regression Coefficients^a^  of the Influence of Humanic Clues on Switching Restraint** | | | | | |
| --- | --- | --- | --- | --- | --- |
|  | Standardized Coefficients | | df | F | Sig. |
|  | Beta | Bootstrap (1000) Estimate of Std. Error |  |  |  |
| The friendly actions of the health care service providers. | .284 | .057 | 4 | 25.085 | .000* |
| The caring expression of the health care service providers. | .290 | .108 | 4 | 7.273 | .000* |
| The tone of voice of the health care service provider. | .341 | .041 | 5 | 67.889 | .000* |
| The body language of the health care service provider. | .320 | .046 | 4 | 47.400 | .000* |
| The respect and courtesy from the health care service provider. | -.006 | .018 | 2 | .102 | .903 |
| Dependent Variable: Switchingrestraint | | | | | |
| * Significant predictors | | | | | |

*Source: Researcher’s Field Survey, 2016*

The result of the model in Table 4.3.3c, shows that the tone of voice of the healthcare service provider having the highest beta value of (*β* = .341) contribute mostly to explaining the influence of humanic clues on switching restraints of healthcare services. This is followed by the body language of the healthcare service provider (*beta* = .320), the caring expression of the service providers (*beta* = .290) and the friendly actions of the service providers scale (*beta* = .284). This means that the healthcare customers of the organisations believe that the tone of voice of the healthcare service provider makes the strongest unique contribution to explaining switching restraint of customers.

**Table 4.3.3: The effects of humanic clues on switching restraint of customers in the healthcare service firms in Nigeria (Healthcare Experts Perspective)**

Table 4.3.3d shows the combined effect of the independent variables (The tone of voice of the health care service provider, the health care service providers are friendly, the behaviour of the health care service provider is consistently positive, the health care service providers are consistently caring) on switching restraint (the dependent variable) of healthcare service customers. Based on the result (Table 4.3.3d), the Table gives the R^2^value to be 0.779 indicating that the total contribution or influence made by all the independent variables is 77.9%. This signifies that the humanic clues had significant joint influence on the switching restraint of healthcare service customers.

| **Table 4.3.3d: Model Summary** | | | |  |
| --- | --- | --- | --- | --- |
| Multiple R | R Square | Adjusted R Square | Apparent Prediction Error | |
| .883 | .779 | .762 | .221 | |
| 1. Predictors: (Constant), The tone of voice of the health care service provider, The health care service providers are friendly, The behaviour of the health care service provider is consistently positive, The health care service providers are consistently caring 2. Dependent Variable: Switchingrestraint   *Source: Researcher’s Field Survey, 2016*  The ANOVA of the regression model is also presented in Table 4.3.3e. It is evident from the result (Table 4.3.3e) that the regression model has F (9,114) = 44.646 is significant with P-value 0.005. This means that the null hypothesis is rejected as we accpt the alternate hypothesis. Again, the result in the Table 4.3.3e further establishes that the composite influence of the functional clues did not occur by chance as it gives the F-ratio value of 44.646 which signifies the strength of the three independent variables (under the humanic clues) as potent predictors of switching restraint of healthcare service customers. | | | |  |

| **Table 4.3.3e: ANOVA** | | | | | | |
| --- | --- | --- | --- | --- | --- | --- |
|  | | Sum of Squares | Df | Mean Square | F | Sig. |
|  | Regression | 96.595 | 9 | 10.733 | 44.646 | .000 |
|  | Residual | 27.405 | 114 | .240 |  |  |
|  | Total | 124.000 | 123 |  |  |  |
| 1. Predictors: (Constant), Humanic clues | | | | | | |
| 1. Dependent Variable: Switchingrestraint | | | | | | |

*Source: Researcher’s Field Survey, 2016*

| **Table 4.3.3f:Coefficients** | | | | | |
| --- | --- | --- | --- | --- | --- |
|  | Standardized Coefficients | | Df | F | Sig. |
|  | Beta | Bootstrap (1000) Estimate of Std. Error |  |  |  |
| The behaviour of the health care service provider. | .294 | .171 | 2 | 2.952 | .056 |
| The health care service providers are friendly | .012 | .120 | 1 | .010 | .920 |
| The health care service providers are consistently caring | .317 | .193 | 3 | 2.698 | .049* |
| The tone of voice of the health care service providers. | .486 | .231 | 3 | 4.442 | .005* |
| Dependent Variable: switching restraint | | | | | |
| * Significant predictors | | | | | |

*Source: Researcher’s Field Survey, 2016*

The result in the Table 4.3.3f shows the healthcare experts opinion on the influence of humanic clues on switching restraint. The result (Table 4.3.3f) reveals that the tone of voice of the healthcare service provider had more statistical significance in predicting switching restraint of customers, recording the highest beta value of (β = .486) than other variables: the healthcare service provider consistent caring (*beta* = .317). This means that the healthcare experts are of the opinion that the tone of voice of the healthcare service provider makes the strongest unique contribution to explaining switching restraint of customers.

## **Test of Hypothesis 4**

The fourth hypothesis was formulated based on the research question and research objective

**Research Question 4**

Is there any significant relationship between customer experience and customer satisfaction in the health care sector in Nigeria?

**Research Objective 4**

To examine the relationship between customer experience and customer satisfaction in the health care sector in Lagos, Nigeria

Derived from the above research question and research objective is the stated hypothesis in null form

**Research Hypothesis 4**

**H_o_**: There is no significant relationship between customer experience and customer satisfaction.

In order to test for the trend and strength of the relationships between the two variables in hypothesis four above, Categorical Regression (CATREG) analysis was employed to find out the degree of relationship that exists between customer experience and customer satisfaction.

| Table 4.3.4a: The R Square tells how much of the variance in the dependent variable (customer satisfaction) is described by the model (Customer experience: The reliability of services; the right diagnosis of illness; the right treatment of illness; the effective verbal communication and the non-verbal communication). Here, the R Square value .555 is stated as a percentage; this means that the model (Customer experience) explains 55.5% of the variance on customer satisfaction. This is very reasonable result when compare to some outcome that are reported in the journals  **Table 4.3.4a: Model Summary of the Relationship between Customer Experience and Satisfaction** | | | | |
| --- | --- | --- | --- | --- |
| Multiple R | | R Square | Adjusted R Square | Apparent Prediction Error |
| .745 | | .555 | .535 | .445 |

Predictors: The reliability of the health care services; the right diagnosis of illness by the health care organisation; the right treatment of illness by the health care organisation; the effective verbal communication of the health care service and he non-verbal communication of the health care service.

Dependent Variable: Customer satisfaction

The ANOVA of the regression model is also presented Table 4.3.4b. It is evident from the result (Table 4.3.4b) that the regression model has F (16,348) = 27.176 is significant with P<0.0005. This means that the null hypothesis (H_0_) is rejected as we accept the alternate hypothesis.

| **Table 4.3.4b: ANOVA of the Relationship between Customer Experience and Satisfaction** | | | | | | |
| --- | --- | --- | --- | --- | --- | --- |
|  | | | | | | |
|  | Sum of Squares | Df | Mean Square | F | Sig. | |
| Regression | 202.740 | 16 | 12.671 | 27.176 | .000 | |
| Residual | 162.260 | 348 | .466 |  |  | |
| Total | 365.000 | 364 |  |  |  | |
| 1. Predictors: (Constant), Customerexperience | | | | | | |
| 1. Dependent Variable: Customersatisfaction   *Source: Researcher’s Field Survey, 2016* | | | | | | |

| **Table 4.3.4c: Coefficients of Regression Analysis of the Relationship between Customer Experience and Satisfaction** | | | | | |
| --- | --- | --- | --- | --- | --- |
|  | Standardized Coefficients | | Df | F | Sig. |
|  | Beta | Bootstrap (1000) Estimate of Std. Error |  |  |  |
| The reliability of the health care services. | .196 | .093 | 3 | 4.432 | .004* |
| The right diagnosis of illness by the health care organisation. | .260 | .095 | 4 | 7.488 | .000* |
| The right treatment of illness by the health care organisation. | .135 | .221 | 3 | 1.136 | .034* |
| The effective verbal communication of the health care service provider. | .153 | .105 | 3 | 2.125 | .097 |
| The non-verbal communication of the health care service provider. | .248 | .088 | 3 | 7.986 | .000* |
| Dependent Variable: Customersatisfaction | | | | | |

*Significant predictors

*Source Researcher’s Field Survey, 2016*

The Table 4.3.4c shows the coefficients of the regression analysis of the relationship between customer experience and satisfaction by customers of the four hospitals investigated. The result in Table 4.3.2c reveals that four factors namely: the right diagnosis of illness, the non-verbal communication of the health care service provider, the reliability of the health care services and the right treatment of illness emerged as the aspects of customer experience that have positive relationship with customer satisfaction. Furthermore, based on the beta values of those predictors as shown in Table 4.3.4c. It is evident that right diagnosis of illness by the health care organisation with the highest beta value of (*β* = .260) contributes most in the relationship between customer experience and satisfaction. This is followed by non-verbal communication of the health care service provider (*beta* = .248), the reliability of the health care services (*beta* = .196) and the right treatment of illness by the health care organisation (*beta* = .135). This means that the healthcare customers believe that the right diagnosis of illness by the health care organisation makes the strongest unique contribution in predicting customer satisfaction.
